# Computational and genetic evidence that different structural conformations of a non-catalytic region affect the function of plant cellulose synthase

**DOI:** 10.1093/jxb/eru383

**Published:** 2014-09-26

**Authors:** Erin Slabaugh, Latsavongsakda Sethaphong, Chaowen Xiao, Joshua Amick, Charles T. Anderson, Candace H. Haigler, Yaroslava G. Yingling

**Affiliations:** ^1^Department of Crop Science and Department of Plant and Microbial Biology, North Carolina State University, Raleigh, NC 27695, USA; ^2^Department of Materials Science and Engineering, North Carolina State University, Raleigh, NC 27695, USA; ^3^Department of Biology, the Pennsylvania State University, University Park, PA 16802, USA

**Keywords:** Cellulose biosynthesis, CESA, genetic complementation, protein structural modelling, *rsw1* mutant, transmembrane helix.

## Abstract

The β-1,4-glucan chains comprising cellulose are synthesized by cellulose synthases in the plasma membranes of diverse organisms including bacteria and plants. Understanding structure–function relationships in the plant enzymes involved in cellulose synthesis (CESAs) is important because cellulose is the most abundant component in the plant cell wall, a key renewable biomaterial. Here, we explored the structure and function of the region encompassing transmembrane helices (TMHs) 5 and 6 in CESA using computational and genetic tools. *Ab initio* computational structure prediction revealed novel bi-modal structural conformations of the region between TMH5 and 6 that may affect CESA function. Here we present our computational findings on this region in three CESAs of *Arabidopsis thaliana* (AtCESA1, 3, and 6), the *Atcesa3* ^*ixr1-2*^ mutant, and a novel missense mutation in AtCESA1. A newly engineered point mutation in AtCESA1 (*Atcesa1* ^*F954L*^) that altered the structural conformation *in silico* resulted in a protein that was not fully functional in the temperature-sensitive *Atcesa1* ^*rsw1-1*^ mutant at the restrictive temperature. The combination of computational and genetic results provides evidence that the ability of the TMH5–6 region to adopt specific structural conformations is important for CESA function.

## Summary

Computational modelling of peptide structure, genetic complementation in *Arabidopsis thaliana*, and confocal microscopy provide evidence that a region between two transmembrane helices may adopt two predominant structural conformations that affect the function of plant cellulose synthase.

## Introduction

Cellulose is an essential structural component of plant cell walls, which are renewable biomaterials used in the manufacture of many products. Cellulose is produced by land plants, as well as by some algae, bacteria, tunicates, and protists. Cellulose is a linear polysaccharide composed of β-1,4 linked glucan chains that are synthesized by cellulose synthases, called CESAs in plants. In plants, multiple β-1,4 linked glucan chains coalesce into microfibrils, which are then incorporated into the cell wall. Cellulose synthases are membrane-bound inverting glycosyltransferases within glycosyltransferase family 2 (GT-2) (Cantarel *et al.*, 2009), and they contain a conserved GT-A fold (Charnock and Davies, 1999). In both plants and bacteria, the β-1,4 linked glucan must be transported outside the cell (Valla *et al.*, 2009; Saxena and Brown, 2012), a process that remains poorly understood in plants.

Seed plants contain a CESA protein family with six distinct types of isoforms, including three required for the synthesis of each of the major types of cell walls. For example, in the model plant *Arabidopsis thaliana*, AtCESA1, 3, and 6 are associated with cellulose synthesis in expanding primary walls, whereas AtCESA4, 7, and 8 participate in secondary wall formation (Desprez *et al.*, 2007; Persson *et al.*, 2007; Tanaka *et al.*, 2003). In each case, the three CESA isoforms are integrated into a cellulose synthesis complex (CSC) that moves in the plane of the plasma membrane as cellulose fibrils are formed (Paredez *et al.*, 2006; Endler and Persson, 2011).

Although no plant CESA has been crystallized, a computational model was generated of the cytosolic domain of GhCESA1 from cotton (Sethaphong *et al.*, 2013). This predicted structure contains a GT-A fold similar to the one in the recently solved crystal structure of BcsA/B from *Rhodobacter sphaeroides* (Morgan *et al.*, 2013). These new cellulose synthase structures have allowed insights into possible mechanistic effects of CESA missense mutations (Morgan *et al.*, 2013; Sethaphong *et al.*, 2013; Slabaugh *et al*., 2013) and may enable structurally informed engineering of cellulose synthase function.

As determined from the recently solved crystal structure of BcsA/B from *Rhodobacter sphaeroides*, the BcsA cellulose synthase contains eight transmembrane helices (TMH) and a large cytosolic domain located between TMH4 and 5 (Morgan *et al.*, 2013). The BcsA/B heterodimer was co-crystallized with a putative short glucan chain bound within a central pore formed by TMH3-8 of BcsA, showing that BcsA synthesizes β-1,4 linked glucan while simultaneously transporting it across the membrane (Morgan *et al.*, 2013).

**Fig. 1.**
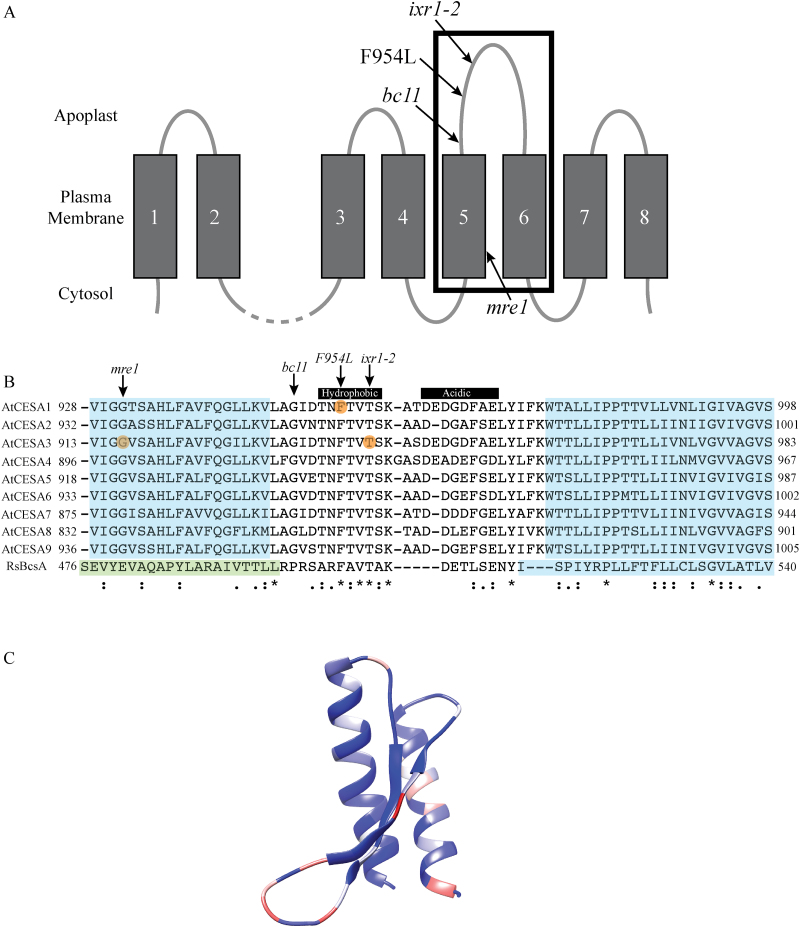
Schematic of the predicted topology of plant CESAs and protein sequence alignment of the computationally modelled region. (A) Plants CESAs are predicted to have eight transmembrane helices (TMHs) with the cytosolic domain between TMH2 and 3, which contains the active site. The TMH5–6 region, which was modelled, is shown within the black box, including the two TMHs and the amino acids between them. Missense mutations within this region are highlighted with arrows. *bc*=brittle culm, *ixr*=isoxaben resistant, *mre*=multiple response expansion. (B) Protein sequence alignment of the modelled TMH5–6 region of AtCESA1, 3, 6 and BcsA is shown, along with homologous sequences of AtCESA2, 4, 5, 7, 8, and 9. Orange circles indicate the *mre1*, F954L, and *ixr1-2* missense mutations. Blue shading indicates TMHs and green shading represents IF3 in BcsA. (C) A snapshot of GhCESA1 coloured to represent protein sequence conservation based on results from a BLAST search of amino acids 820–890. Blue indicates 100% sequence conservation, white indicates 72% conservation, and red indicates 30% sequence conservation. The snapshot was generated using Chimera software (Pettersen *et al.*, 2004). For a list of protein sequences used to create this snapshot, please see Supplementary Table S2.

Despite similarities in the structure and function of plant CESAs and bacterial BcsA, there are substantial differences between the proteins. For example, only CESA contains an N-terminal zinc finger domain and two ~125 amino acid insertions in the cytosolic domain (Pear *et al.*, 1996), whereas only BcsA contains a C-terminal PilZ domain that is regulated by cyclic-di-GMP (c-di-GMP) (Morgan *et al.*, 2013). In addition, the predicted membrane topology of plant CESAs is not consistent with BcsA. Similar to BcsA, plant CESAs are predicted to contain eight TMHs (Fig. 1A) (Jones 1999; Schwacke *et al.*, 2003; Viklund and Elofsson, 2008). However, the catalytically active cytosolic domain lies between TMH2 and 3 of CESAs and between TMH4 and 5 of BcsA (Morgan *et al.*, 2013; Sethaphong *et al.*, 2013). Additionally, BcsA contains a cytosolic interfacial helix (IF3) between TMH6 and 7, which is not predicted in plant CESAs by membrane topology prediction algorithms.

Mutational evidence has been used to argue for a role of the plant transmembrane region, inclusive of TMH5 and 6, in the organization of the nascent cellulose fibril (Harris *et al.*, 2012) or proper CESA trafficking (Zhang *et al.*, 2009). Although we do not know its topology, three missense mutations in the TMH5–6 region of CESA (Fig. 1A, B) imply that it is functionally important for plant cellulose synthesis. The *mre1* mutation is a G916E substitution within TMH5 of AtCESA3 that results in sensitivity to an ethylene inhibitor (aminoisobutyric acid, AIB), an expanded root when grown on high sucrose medium, slightly reduced growth of the rosette and inflorescence stem, and short roots with reduced cellulose (Pysh *et al.*, 2012). The *bc11* mutation is a G858R mutation in OsCESA4 (homologous to AtCESA8) that results in a brittle culm, dwarfing, increased arabinoxylan to compensate for reduced cellulose, and resistance to a cellulose-synthesis inhibitor (2,6-dichlorobenzonitrile, DCB) (Zhang *et al.*, 2009). The *ixr1-2* mutation, which is a T942I substitution in CESA3, results in resistance to the cellulose-synthesis inhibitor isoxaben, reduced cellulose microfibril crystallinity, and increased CESA velocity at the plasma membrane (Scheible *et al.*, 2001; Harris et al 2012).

Despite evidence for the functional importance of the TMH5–6 region in CESA, there was previously no mechanistic insight into how the existing missense mutations exert their effects. Here we report a combined computational and genetic approach to obtain more insights into structure–function relationships in the TMH5–6 region. We generated *ab initio* computational models of the TMH5–6 region in wild-type and mutant *Arabidopsis* CESAs. Based on *in silico* evidence of two potential conformations for the linker between TMH5 and 6, we engineered a novel missense mutation in AtCESA1 that altered the energy barrier between the two different structural conformations and assessed its effect on CESA function through phenotypic complementation of the temperature-sensitive *rsw1-1* mutation in AtCESA1 (*Atcesa1* ^*rsw1*^), which causes short roots when plants are grown at the restrictive temperature (Arioli *et al.*, 1998). Taken together, these data provide evidence that CESA function is influenced by the ability of the amino acids between TMH5 and 6 to adopt specific conformational states.

## Materials and methods

### Simulations and modelling

Secondary structure predictions were generated using Psipred (Jones, 1999) and the topology prediction of the TMH region was obtained with the OCTOPUS software utility (Viklund and Elofsson, 2008). Lipophilicity scoring was used to further constrain the predictions (Yarov-Yarovoy *et al.*, 2006), and additional constraints for Rosetta were obtained from the JUFO utility (Meiler and Baker, 2003). Starting coordinates of the amino acid sequence in linear form were generated using the tleap utility of the molecular dynamics software suite AMBER. An in-house script cleaned the resulting coordinate file to be compatible with ROSETTA atom naming conventions.

Three-dimensional structural decoys of the TMH5–6 regions of native and mutant CESAs were generated using the membrane protein folding algorithm of ROSETTA (ver 3.4) (Yarov-Yarovoy *et al.*, 2006) (see Supplementary Table S1 for details). The ROSETTA fragment library database used was generated using the 2010 NCBI non-redundant protein database. Optimal structures were relaxed using the ROSETTA *ab initio* relax method under a weak constraint. As the decoys resulting from production runs did not form a clear folding plot that converged on a low-REU decoy, clustering analysis was employed to select decoys predicted to be near-native (energetically stable) structures. The Durandal algorithm and maximum-entropy based clustering of the top ten percent best scoring decoys were used for the selection of model structures (Berenger *et al.*, 2012).

Origin (OriginLab, Northampton, MA) or Tecplot (Tecplot Inc., Bellevue, WA) were used to generate the folding plots and PyMOL (The PyMOL Molecular Graphics System, Version 1.2r3pre, Schrödinger, LLC) and Discovery Studio (Accelrys Software Inc., San Diego, CA) were used to view and render protein structures. Additional folding plots were generated using a custom shell script and the Durandal software utility that calculated the C-alpha root-mean-square deviation (CARMSD) between all generated decoys against the best scoring structure. ROSETTA does have a scoring utility, but it is significantly slower than Durandal. To obtain decoy densities, frequency counts needed for heatmaps were generated by binning in areas of 0.5 REU by 1 Å CARMSD. Structures of the region of BcsA corresponding to the TMH5–6 region of plant CESA were taken from the crystal structure (PDB ID: 4HG6) and rendered with PyMOL. A custom batch script that leveraged local installations of all the required utilities and topology prediction engines except for JUFO was created to accomplish this process of linear sequence to initial analysis and automated structure selection.

### Molecular cloning and *Arabidopsis* transformation

The coding sequence (CDS) of CESA1 was obtained from TAIR (www.Arabidopsis.org). Site-directed mutagenesis was performed using overlap PCR to generate the F954L mutation. Primer sequences for mutagenesis that contained a TTC to TTG mutation were: forward primer: GGT ATC GAC ACC AAC TT*G* ACC GTT ACA TC; reverse primer: GAT GTA ACG GT*C* AAG TTG GTG TCG ATA CC. Foreign gene expression was driven by the native promoter of CESA1. The native promoter sequence, including the 5′ UTR and 1kb of upstream sequence, was obtained from PlantPromoterDb (http://ppdb.agr.gifu-u.ac.jp/ppdb/cgi-bin/index.cgi). The native promoters and CDSs were cloned into the pFGC5941 binary vector (TAIR: CD3-447), using restriction digest with StuI/XhoI to insert the promoter and AscI/SwaI to insert the CDS. The cauliflower mosaic virus 35S promoter was removed from pFGC5941 using StuI/Xho1 sites. Constructs were transformed into *Agrobacterium tumefaciens* strain GV3101. Stable transformation of *Arabidopsis* was carried out using the floral dip method (Clough and Bent, 1998).

### Plant materials and growth conditions

*Arabidopsis* seeds were surfaced sterilized by washing for 30 s each with 10% bleach, twice in sterile water, 70% ethanol, then twice in sterile water. Seeds were completely dried on filter paper and vernalized for at least 24h at 4 °C before plating on half-strength Murashige and Skoog medium with 1% (w/v) sucrose and 0.4% (w/v) Phytagel^TM^ (Sigma Aldrich; St. Louis, MO). *Arabidopsis* plants were grown at 23 °C in a 16h photoperiod under illumination of 100 µmol m^–2^ s^–1^ at 50% relative humidity.

### Phenotyping Atcesa1^rsw1^ and Atcesa6^prc1^ Atcesa1^rsw1^ YFP–CESA6 lines

Seeds were grown on vertical plates at 23 °C (permissive temperature) for 5 d, then transferred to 31 °C (restrictive temperature) for an additional 7 d. Seedlings were grown under continuous light of approximately 100 µmol m^–2^ s^–1^ and 50% relative humidity. Primary roots were photographed then analysed using ImageJ (http://rsbweb.nih.gov/ij/). The means for root length were derived from measurements of 10–30 individual seedlings per line from at least two independent experiments.

### Confocal microscopy

*Atcesa6* ^*prc1*^ *Atcesa1* ^*rsw1*^ *YFP–CESA6* and *Atcesa6* ^*prc1*^ *Atcesa1* ^*rsw1*^ *YFP–CESA6 Atcesa1* ^*F954L*^ seeds were surface-sterilized in 30% bleach + 0.1% (w/v) SDS for 20min, washed four times with sterile water, stored at 4 °C for 2 d, and sown on half-strength Murashige and Skoog medium without sucrose. Plates were exposed to light for 2h to stimulate germination, then wrapped in two layers of aluminium foil and grown vertically for 3 d at 22 °C. For restrictive temperature treatment, plates with three-day-old etiolated seedlings were transferred to a 29 °C incubator for 24h. For all genotypes, YFP fluorescence was detected in hypocotyl cells just below the apical hook using a 100 mW 488nm excitation laser at 30% power and a 525/50nm emission filter on a Zeiss Observer SD spinning disk confocal microscope with a ×100 1.40 NA oil-immersion objective and a Photometrics QuantEM 512SC camera (exposure time=400ms, EM gain=1000, readout gain=1). YFP–CESA6 particle density for z series was quantified computationally using the Spot Detection function of Imaris (Bitplane) (Chen *et al.*, 2010). For fluorescence intensity measurements, z series were opened in ImageJ, extraneous z slices were removed using the Substack Maker plugin, maximum projections were generated and background subtracted using a sliding paraboloid radius of 10 pixels, and Integrated Density for the entire image was measured.

## Results

### Computational modelling of TMH5–6 in *Arabidopsis* CESA1, 3, and 6

Protein sequence alignment of the region between TMH5–6 indicated high conservation in length, hydrophobicity, and acidity (Fig. 1B, C and Supplementary Table S2); TMH5–6 is the longest predicted TMH linker (27–29 amino acids) found among CESAs and is predicted to localize to the apoplast in the conventional eight TMH model (Fig. 1A). The hydrophobic segment of the linker is well-conserved, but the acidic side is less so, owing to aspartic acid substitution for glutamic acid (Fig. 1B).

**Fig. 2.**
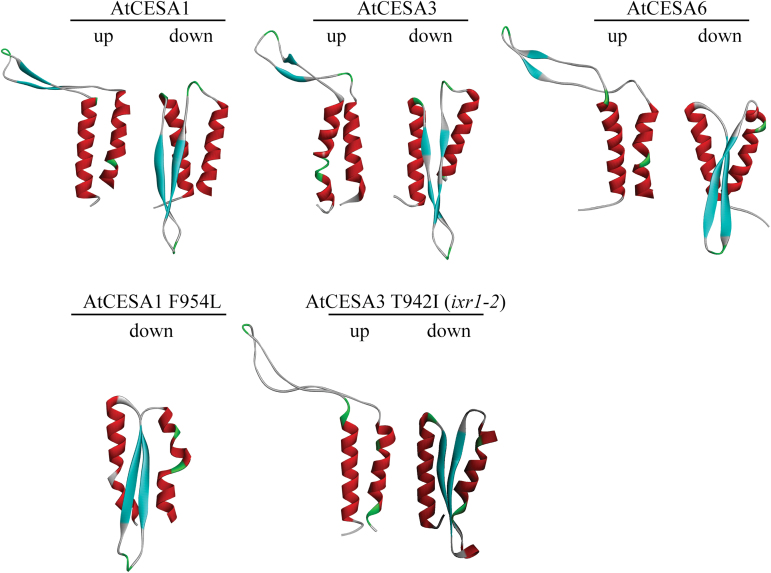
Computationally predicted structural conformations of the TMH5–6 region of wild-type AtCESA1, 3, 6, and the Atcesa1^F954L^ and Atcesa3^ixr1-2^ (T942I) mutants. Models shown are representative of the “up” or “down” structural conformations. The “up” conformation indicates that the intervening region between TMH5 and 6 is extended away from the helices, whereas it forms a β-hairpin structure parallel to the axes of the helices in the “down” conformation. For all decoys, TMH5 is oriented on the left-hand side and TMH6 is on the right-hand side.

*Ab initio* computational models of the region encompassing TMH5–6 (Fig. 1A) of CESAs from *Arabidopsis* (Fig. 2) were generated using ROSETTA (Yarov-Yarovoy *et al.*, 2006; Vinothkumar and Henderson, 2010). The largest population of atomistic model structures, known as decoys, were scored as lower than zero Rosetta Energy Units (REU), which corresponds to 0.5 kcal mol^–1^ Gibb’s free energy, indicating allowable energetics for the modelled structures (Das, 2011). Folding plots of the decoys were generated by plotting REU against the conformational space in C-alpha root-mean-square deviation (CARMSD) in angstroms (Å). The biphasic nature of the folding plots (Supplementary Fig. S1) suggests that the TMH5–6 linker exhibits conformational entropy (Arkun and Erman, 2010) within which two structures may dominate: (i) an extended conformation (called hereafter the “up” conformation); or (ii) a β-hairpin structure parallel to the helical axes of TMH5 and 6 (called hereafter the “down” conformation) (Fig. 2). Given the small energy barrier between these two conformations (approximately 10 kcal mol^–1^), the linker might adopt either conformation *in vivo*. Although folding only two of the eight predicted TMHs presents an underconstrained problem, the small energy difference between the “up” and “down” conformations suggests that there may be a binary state for this region.

To validate our *ab initio* method, we generated a computational model of the region between IF3 and TMH7 from BcsA and compared it to the published crystal structure (Morgan *et al.* 2013). This region in BscA (Supplementary Fig. S2A) functions as a gating loop that regulates substrate access into the active site (Morgan *et al.*, 2014). The computational model of amino acids 476–540 of BcsA showed a pair of helices and a long coil (Supplementary Fig. S2B, C). Structural alignment showed that the model was similar to the crystal structure (Supplementary Fig. S2D). However, the model of the IF3–TMH7 region of BcsA did not show a biphasic folding plot (Supplementary Fig. S3), as was observed for wild-type AtCESA1, 3, and 6.

### Computational modelling of TMH5–6 containing the Atcesa3^ixr1-2^ and Atcesa1^F954L^ mutations

To explore how missense mutations between TMH5 and 6 may affect CESA function, we compared structural models and folding plots of mutants with those of wild type. Any altered distribution of decoys within the folding plots correlates with a possibility that the preferred structural conformation of the mutant protein is altered relative to wild-type. Computational models of a CESA3 TMH5–6 region containing the T942I (*ixr1-2*) missense mutation (Scheible *et al.*, 2001) (Fig. 2) showed a biphasic folding plot similar to wild type (Supplementary Fig. S1), suggesting that this mutation does not alter the occurrence of two distinct structural conformations. However, more decoys in the largest cluster are located in the region that corresponds to the “up” conformation compared with wild-type AtCESA3, suggesting that the T942I mutation promotes the “up” conformation (Supplementary Fig. S1). Possibly, this structural change hinders interaction with the isoxaben herbicide as well as altering AtCESA3 function in a way that ultimately reduces cellulose crystallinity (Harris *et al.*, 2012).

The F954 residue is highly conserved among plant and bacterial cellulose synthases (Fig. 1B) and is likely to be important for CESA function. To test a potentially more marked alteration of the structure of the TMH5–6 region, we generated a F954L missense mutation in AtCESA1 that replaced the native hydrophobic phenylalanine residue with the smaller hydrophobic leucine residue. The F954L mutation changed the predicted structure of the linker between TMH5 and 6 (Fig. 2) and eliminated the bimodal distribution in the computational folding plot (Supplementary Fig. S1). The mutation also resulted in a redistribution of decoys in the largest cluster that favours structures in the “down” conformation, compared with wild-type AtCESA1 (Supplementary Fig. S1).

### The Atcesa1^F954L^ mutant protein is non-functional in *Arabidopsis*

**Fig. 3.**
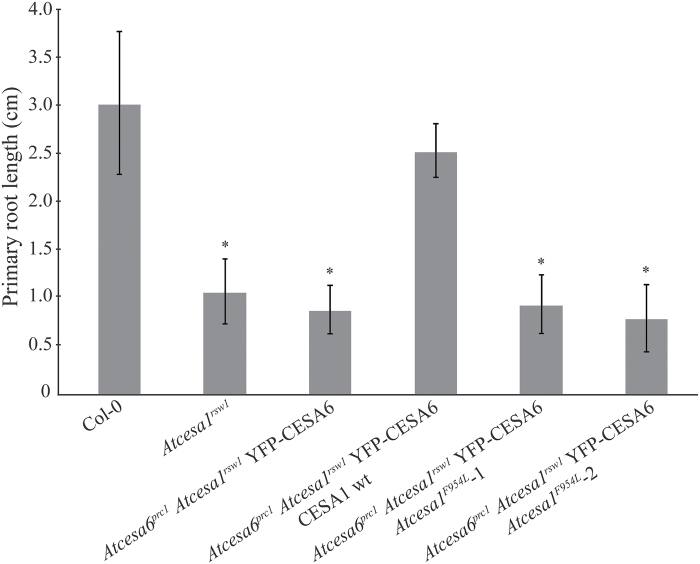
The *Atcesa1* ^*F954L*^ mutation does not rescue the short root phenotype of the *Atcesa6* ^*prc1*^ *Atcesa1* ^*rsw1*^ *YFP–CESA6* mutant. Seedlings were grown for 5 d at a permissive temperature (23 °C), and then transferred to a restrictive temperature (31 °C) for 7 d. After the *rsw1* mutation in *Atcesa6* ^*prc1*^ *Atcesa1* ^*rsw1*^ *YFP–CESA6* was rescued with wild-type CESA1, root length was similar to Col-0 because wild-type CESA1 replaced the *rsw1* mutant protein. As expected, *Atcesa1* ^*rsw1*^ roots were short, as were roots of *Atcesa6* ^*prc1*^ *Atcesa1* ^*rsw1*^ *YFP–CESA6* seedlings that contained only the *rsw1* allele of AtCESA1. Two independent *Atcesa6* ^*prc1*^ *Atcesa1* ^*rsw1*^ *YFP–CESA6* lines in which *Atcesa1* ^*F954L*^ was expressed (*Atcesa6* ^*prc1*^ *Atcesa1* ^*rsw1*^ *YFP–CESA6 Atcesa1* ^*F954L*^-1 and -2) also had short roots, indicating that AtCESA1 is non-functional when the F954L mutation is present. Total root length was measured, error bars represent standard deviation, and asterisks indicate significant differences (*P*<0.0001, t-test) compared with wild-type Col-0.

To test whether the F954L mutation affects CESA1 function *in vivo*, we generated stable *Arabidopsis* transformants in the *Atcesa1* ^*rsw1*^ and *Atcesa6* ^*prc1*^ *Atcesa1* ^*rsw1*^ *YFP–CESA6* backgrounds. The *rsw1-1* mutant is characterized by an A549V substitution in CESA1. The *Atcesa6* ^*prc1*^ *Atcesa1* ^*rsw1*^ *YFP–CESA6* line also contains the *rsw1-1* allele, but the *prc1* mutation in AtCESA6 has been complemented with a fluorescently tagged CESA6 to allow for tracking of CSCs (containing AtCESA1, 3, and 6) using confocal microscopy. As the *rsw1* allele hinders root growth at restrictive temperatures (29–31 °C) in both lines as compared with the permissive temperature (22–23 °C) (Arioli *et al.*, 1998; Chen *et al.*, 2010), we were able to assess CESA function by analysing root length at 31 °C. When *Atcesa1* ^*F954L*^ was expressed in the *Atcesa6* ^*prc1*^ *Atcesa1* ^*rsw1*^ *YFP–CESA6* line, short roots developed at a restrictive temperature in contrast to long roots in wild-type plants or mutant lines complemented with wild-type AtCESA1 (Fig. 3). This result supported the non-functionality of Atcesa1^F954L^, assuming that the mutant protein was present.

Confocal microscopy was used in an indirect assay to confirm that the Atcesa1^F954L^ protein was being expressed in the experimental lines. An indirect method was required because a mutant Atcesa1^rsw1^ protein (with only one amino acid change compared with wild-type) is present in the *Atcesa6* ^*prc1*^ *Atcesa1* ^*rsw1*^ *YFP–CESA6* line, prohibiting the use of immunoblotting to confirm the presence of Atcesa1^F954L^ in the transformed experimental line. Previous research (Fujita *et al.*, 2013; Chen *et al.*, 2010) has shown that under restrictive temperatures, YFP–CESA6 fluorescence is largely absent from the plasma membrane and is detected mainly in endomembrane compartments of *Atcesa6* ^*prc1*^ *Atcesa1* ^*rsw1*^ *YFP–CESA6* mutant seedlings. In contrast, YFP–CESA6 exists in both the plasma membrane and endomembrane compartments at restrictive temperatures when a wild-type copy of CESA1 is expressed in the *Atcesa6* ^*prc1*^ *Atcesa1* ^*rsw1*^ *YFP–CESA6* background. These prior results, which reflect the co-existence of AtCESA1, 3, and 6 in functional CSCs in the plasma membrane, are diagrammed in Supplementary Fig. S4 to aid the interpretation of our results on expression of the *Atcesa1* ^*F954L*^ mutant allele.

**Fig. 4.**
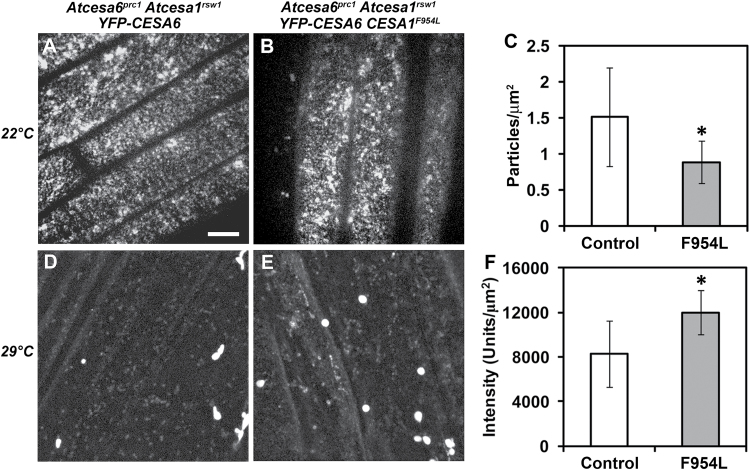
Confocal images of YFP–CESA6 in control and mutant lines at permissive (22 °C) and restrictive (29 °C) temperatures. (A, B) At 22 °C, YFP–CESA6 predominantly localizes to punctae in the plasma membrane in *Atcesa6* ^*prc1*^ *Atcesa1* ^*rsw1*^ *YFP–CESA6* and *Atcesa6* ^*prc1*^ *Atcesa1* ^*rsw1*^ *YFP–CESA6 Atcesa1* ^*F954L*^ dark-grown hypocotyls. (C) At 22 °C, the density of YFP–CEAS6 particles at the cell surface in the *Atcesa6* ^*prc1*^ *Atcesa1* ^*rsw1*^ *YFP–CESA6 Atcesa1* ^*F954L*^ (F954L) mutant was lower than in the *Atcesa6* ^*prc1*^ *Atcesa1* ^*rsw1*^ *YFP–CESA6* (Control) line. Mean values ±SD are plotted and were derived from 10–11 cells from 7–10 seedlings per line. Asterisk indicates significant difference (*P*<0.05, t-test). (D, E) On exposure to 29 °C for 24h, YFP–CESA6 signal is lower in *Atcesa6* ^*prc1*^ *Atcesa1* ^*rsw1*^ *YFP–CESA6* hypocotyls than in *Atcesa6* ^*prc1*^ *Atcesa1* ^*rsw1*^ *YFP–CESA6 Atcesa1* ^*F954L*^ hypocotyls, where YFP–CESA6 signal is more evident in endomembrane compartments. (F) Quantification of total fluorescence intensity for *Atcesa6* ^*prc1*^ *Atcesa1* ^*rsw1*^ *YFP–CESA6* (Control) and *Atcesa6* ^*prc1*^ *Atcesa1* ^*rsw1*^ *YFP–CESA6 Atcesa1* ^*F954L*^ (F954L) z series collected after exposure to 29 °C for 24h. All images were collected from the same cellular region with identical exposure settings (see Materials and methods) and are representative of *n*≥5 seedlings from three experiments. Displayed images are maximum projections of z series. Scale bar=10 µm. Asterisk indicates significant difference compared with the control (*P*<0.001, t-test).

At the permissive temperature (22 °C), we observed small punctate structures in the plasma membrane of both *Atcesa6* ^*prc1*^ *Atcesa1* ^*rsw1*^ *YFP–CESA6* and *Atcesa6* ^*prc1*^ *Atcesa1* ^*rsw1*^ *YFP–CESA6 Atcesa1* ^*F954L*^ lines (Fig. 4A, B). These typical results for imaging active CESAs in the plasma membrane (Paredez *et al.*, 2006) occurred despite the expression of the potentially disruptive Atcesa1^F954L^ mutant because the *rsw1* allele supports normal CESA function at 22 °C (Arioli *et al.*, 1998; Chen *et al.*, 2010). We also noted that the density of plasma membrane-associated YFP–CESA6 particles in *Atcesa6* ^*prc1*^ *Atcesa1* ^*rsw1*^ *YFP–CESA6 Atcesa1* ^*F954L*^ was lower than in the *Atcesa6* ^*prc1*^ *Atcesa1* ^*rsw1*^ *YFP–CESA6* control line at 22 °C (Fig. 4C), a result that is consistent with impaired functionality of Atcesa1^F954L^ (see Discussion). At the restrictive temperature (29 °C), YFP–CESA6 was almost entirely absent from the plasma membrane in the *Atcesa6* ^*prc1*^ *Atcesa1* ^*rsw1*^ *YFP–CESA6* line (Fig. 4D), as expected. When *Atcesa1* ^*F954L*^ was expressed in the *Atcesa6* ^*prc1*^ *Atcesa1* ^*rsw1*^ *YFP–CESA6 Atcesa1* ^*F954L*^ line, the total YFP–CESA6 signal was significantly stronger at 29 °C as compared with the *Atcesa6* ^*prc1*^ *Atcesa1* ^*rsw1*^ *YFP–CESA6* control line (Fig. 4D–F), but was observed mainly in endomembrane compartments in both lines (Fig. 4D, E). These results are consistent with both the presence and abnormal function of mutant Atcesa1^F954L^ (see Discussion).

## Discussion

The results reported here provide computational and genetic evidence that the structural conformation of the amino acids between TMH5 and 6 affects CESA function. The novel F954L mutation prevents the formation of the extended “up” conformation *in silico* and hinders normal cellulose synthesis *in vivo* as indicated by short roots at the restrictive temperature of two independent *Arabidopsis Atcesa6* ^*prc1*^ *Atcesa1* ^*rsw1*^ *YFP–CESA6 Atcesa1* ^*F954L*^ lines. As the *rsw1* allele is a missense mutation and not a null, confocal microscopy was used to demonstrate that the Atcesa1^F954L^ protein was present in the *Atcesa6* ^*prc1*^ *Atcesa1* ^*rsw1*^ *YFP–CESA6* background even though the *rsw1* short root phenotype was not rescued. In this case, YFP–CESA6 is used as an indicator of CSCs that must also contain AtCESA1 owing to the non-redundant function and close interactions in the plasma membrane of AtCESA1, 3, and 6 during primary wall cellulose synthesis in growing hypocotyls (Lei *et al.*, 2012).

Conveniently, the *rsw1* allele acts as an effective null at the restrictive temperature because mutant CESAs/CSCs disappear from the plasma membrane (Arioli *et al.*, 1998; Chen *et al.*, 2010). This presents the opportunity to attempt to retain CESAs/CSCs in the membrane at the restrictive temperature by expression of AtCESA1 with engineered missense mutations that may or may not affect CESA function. Available evidence suggests that only functional CSCs are transported to and/or retained in the plasma membrane, whereas non-functional CSCs or chemical perturbations of cellulose synthesis often cause CESA accumulation in endomembrane compartments (Taylor *et al*., 2008; Crowell *et al.*, 2009; Gutierrez *et al.*, 2009).

In our experiments to test the effects of the F954L mutation on AtCESA1, the data support both the presence of the AtCESA1^F954L^ protein and lack of functionality of the mutant protein. This is shown by stronger YFP–CESA6 fluorescence (mainly in endomembrane compartments) as compared with the control line when *Atcesa6* ^*prc1*^ *Atcesa1* ^*rsw1*^ *YFP–CESA6 Atcesa1* ^*F954L*^ seedlings were grown at 29 °C (Fig. 4E, F). The AtCESA1^F954L^ protein can partly compensate for the severe depletion of CSCs caused by the temperature-sensitive *rsw1* mutation at 29 °C (Fig. 4D), but the small punctate structures typical of active CSCs in the plasma membrane are sparse compared with the 22 °C permissive temperature (compare images B and E). At 22 °C, a fluorescence pattern typical of active CESAs in the plasma membrane occurred in the *Atcesa6* ^*prc1*^ *Atcesa1* ^*rsw1*^ *YFP–CESA6 Atcesa1* ^*F954L*^ line (Fig. 4B), probably because the AtCESA1^rsw1^ protein was preferentially incorporated into and supported normal CSC behaviour at the permissive temperature. However, the reduced density of CSCs in the plasma membrane as compared with the *Atcesa6* ^*prc1*^ *Atcesa1* ^*rsw1*^ *YFP–CESA6* line grown at 22 °C (Fig. 4A, C) suggests that non-functional AtCESA1^F954L^ protein might have displaced AtCESA1^rsw1^ in some CSCs in the *Atcesa6* ^*prc1*^ *Atcesa1* ^*rsw1*^ *YFP–CESA6 Atcesa1* ^*F954L*^ line and abrogated CSC delivery and/or retention in the plasma membrane at 22 °C.

Residues that are conserved in both plant CESA TMH5–6 and BcsA IF3–TMH7, denoted as an FXVTXK motif, have recently been shown to be important for substrate gating. In BcsA, residues F503 and V505 interact with hydrophobic residues in IF2, IF3, and TMH7 when the gating loop is in the open position (Morgan *et al.*, 2014). When the gating loop is inserted into the active site, residues T506 and K508 probably coordinate the UDP moiety of the substrate, possibly stabilizing UDP during catalysis (Morgan *et al.*, 2014). Although currently available data cannot resolve whether the amino acids between CESA TMH5 and 6 are in the apoplast or the cytosol of plant cells, the important role of the putatively orthologous region in BcsA for gating the substrate binding site (Morgan *et al.*, 2014) resonates with the possibility that the current predictions for CESA topology are incorrect. One possibility that would overcome this topological discrepancy would be for plant CESA TMH3, 4, or 5 to be an interfacial helix and not a membrane-spanning TMH. If this were the case, then the region between TMH5 and 6 in plant CESAs would be exposed to the cytosol, similarly to BcsA, and likewise might function in a similar manner. The alternative *in silico* conformations demonstrated in our results might then be a manifestation of movement of a substrate gating loop in CESA, as supported by crystal structures of BcsA.

Alternatively, the topology of BcsA and CESA might be partly different. The interaction between c-di-GMP and the PilZ domain of BcsA, which regulates the movement of the IF3–TMH7 gating loop (Morgan *et al.*, 2014), represents a mechanism that is not operative for plant CESAs because the PilZ domain and c-di-GMP are not present. It could be that the TMH5–6 region does not serve to regulate substrate access to the CESA catalytic site. This idea is supported by the lack of a biphasic folding plot of computationally modelled IF3–TMH7 in BcsA, compared with the biphasic folding plots of the computational models of TMH5–6 in AtCESA1, 3, and 6 (Supplementary Figs S1, S3). If the predicted topology of plant CESAs proves to be accurate, it may be that plant CESAs have developed different ways to regulate cellulose biosynthesis compared with BcsA, including mechanisms of controlling substrate access to the active site and/or the regulation of translocation and/or deposition of the cellulose microfibril. Although we do not yet know which aspect of CESA function may be affected by the potential *in vivo* differences in conformation of the TMH5–6 region, our results demonstrate an association between altered CESA function *in vivo* and altered protein conformation *in silico*.

## Supplementary data

Supplementary data are available at *JXB* online

Figure S1. Folding plots of AtCESA TMH5–6 region.

Figure S2. Computational models of IF3–TMH7 of BcsA compared with the solved crystal structure of BcsA.

Figure S3. Folding plot of the IF3–TMH7 region from BcsA.

Figure S4. Schematic of the subcellular localization of YFP–CESA6 at restrictive temperatures in *Atcesa6* ^*prc1*^ *Atcesa1* ^*rsw1*^ *YFP–CESA6* seedlings without and with transformation with wild-type AtCESA1.

Table S1. Table of statistical information describing the computational modelling data.

Table S2. The results from a BLAST search of amino acids 820–890 from GhCESA1 (GenBank Accession P93155) were used to create a visual representation of the evolutionary conservation of the protein sequence of the TMH5–6 region from CESAs depicted in Figure 1C.

Supplementary Data

## Abbreviations:

AIB: aminoisobutyric acid
bc11: brittle culm11
BcsA/B: bacterial cellulose synthaseA/B
CARMSD: C-alpha root-mean-square deviation
c-di-GMP: cyclic-di-GMP
CDS: coding sequence
CESA: cellulose synthase
CSC: cellulose synthesis complex
DCB: 2,6-dichlorobenzonitrile
GT-2: Glycosyltransferase Family 2
IF: interfacial helix
ixr1-2: isoxaben resistant1-2
mre1: multiple response expansion1
REU: ROSETTA energy units
rsw1-1: radial swelling1-1
TMH: transmembrane helix
YFP: yellow fluorescent protein.
