# Supplementary material for: Computational and genetic evidence that different structural conformations of a non-catalytic region affect the function of plant cellulose synthase
Source: J Exp Bot. 2014 Sep 26;65(22):6645–53. doi: 10.1093/jxb/eru383 (PMC4246192; doi:10.1093/jxb/eru383)
Supplement: Supplementary Data [file supp_65_22_6645__index.html]

Computational and genetic evidence that different structural conformations of a non-catalytic region affect the function of plant cellulose synthase — Computational and genetic evidence that different structural conformations of a non-catalytic region affect the function of plant cellulose synthase — Supplementary Data 

# Computational and genetic evidence that different structural conformations of a non-catalytic region affect the function of plant cellulose synthase

## Supplementary Data

Data files

**Files in this Data Supplement:**

- Supplementary Data - Supplementary Data
