# Supplementary material for: Computational and genetic evidence that different structural conformations of a non-catalytic region affect the function of plant cellulose synthase
Source: J Exp Bot. 2014 Sep 26;65(22):6645–53. doi: 10.1093/jxb/eru383 (PMC4246192; doi:10.1093/jxb/eru383)
Supplement: Supplementary Data [file supp_eru383_jexbot126052_file001.pdf]

Supplementary data may be found at JXB online

Figure S1. Folding plots of AtCESA TMH5-6 region. Folding plots were generated by plotting REU values of all decoys against the conformational space in RMSD relative to the decoy with the lowest REU value (the "best scoring" decoy, see Table S1). Folding plots of decoys from all TMH5-6 sequences that were modeled showed biphasic distributions except the *Atcesa1*^{F954L} mutant. In the color scale, red indicates areas with the highest density of decoys. The top 10% best scoring structures were used for further clustering of decoys into structurally related groups with less than 6 Å deviation from each other (see Materials and Methods and Table S1). The REU cutoff value, which is the highest REU score among the top 10% best scoring structures, is represented by a black line at approximately -20 REU (shown in the AtCESA1 wt panel). Generally, decoys with RMSD values lower than approximately 9 Å (represented by the lower dashed line in the AtCESA1 wt panel) relative to the best scoring decoy are in the "down" conformation, while decoys with RMSD values between approximately 9 and 17 Å (represented by the dashed lines, respectively) relative to the best scoring decoy are in the "up" conformation.

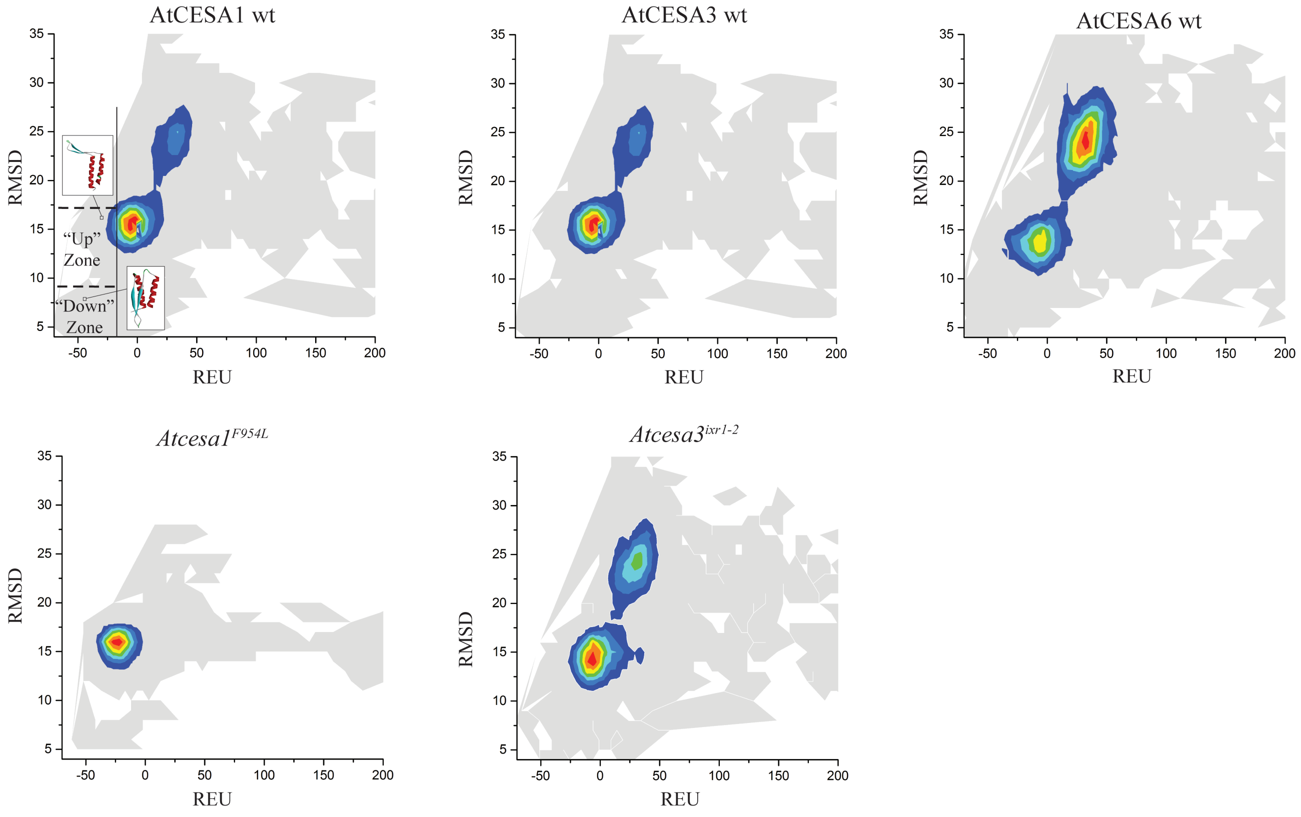

Figure S2. Computational models of IF3-TMH7 of BcsA compared with the solved crystal structure of BcsA. The IF3-TMH7 region includes amino acids 476-540 of BcsA. A) Representation of secondary structure elements of the BcsA crystal structure. The IF3-TMH7 region is highlighted in blue. B, C) Representative decoys of the modeled IF3-TMH7 region of BcsA. D) Structural alignment of the modeled decoys (red and yellow) with the crystal structure of the IF3-TMH7 region of BcsA (blue).

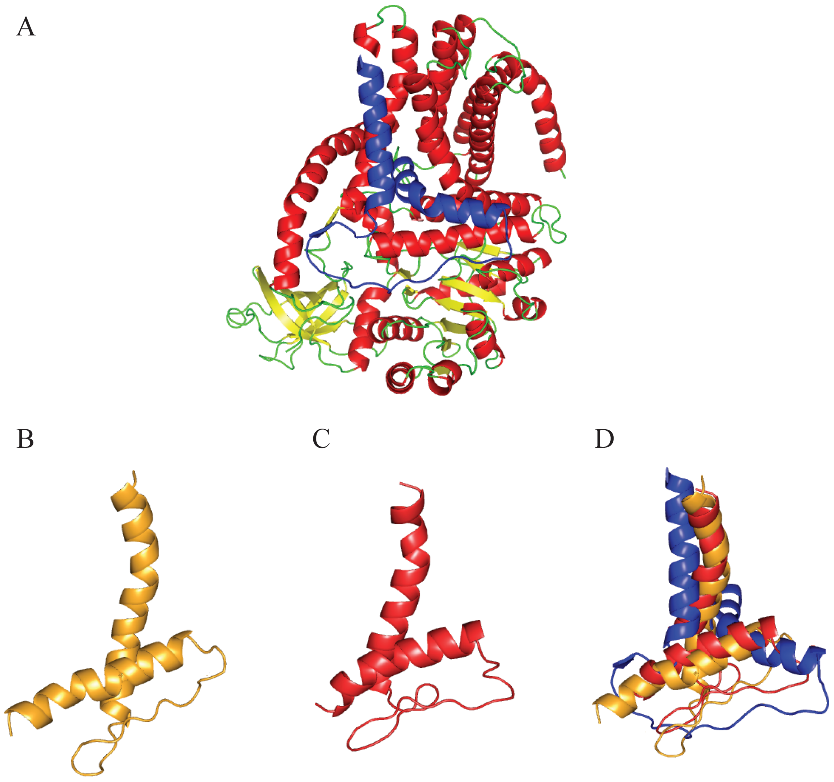

Figure S3. Folding plot of the IF3-TMH7 region from BcsA. The density of decoys in RMSD values relative to the best scoring structure plotted against their REU values generates a folding plot. This plot did not show a biphasic distribution of decoys. Red indicates areas with the highest density of decoys. The grey line indicates the boundary where there is at least one decoy present.

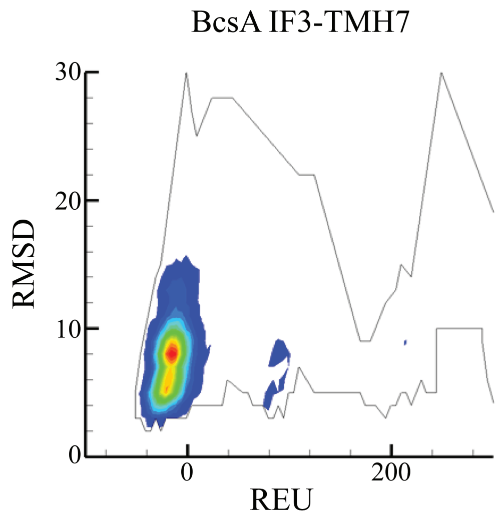

Figure S4. Schematic of the subcellular localization of YFP-CESA6 at restrictive temperatures in *Atcesa6^prc1 Atcesa1^rsw1 YFP-CESA6* seedlings without and with transformation with wild-type AtCESA1. The diagram is based on data in Chen *et al.*, (2010) and Fujita *et al.*, (2013). When only the *rsw1* allele of CESA1 is expressed in the *Atcesa6^prc1 Atcesa1^rsw1 YFP-CESA6* seedlings, exposure to 29°C (the restrictive temperature) results in loss of detectable YFP-CESA6 from the plasma membrane while localization to endomembrane compartments is maintained. When wild-type AtCESA1 is expressed in the *Atcesa6^prc1 Atcesa1^rsw1 YFP-CESA6* background, YFP-CESA6 remains at the plasma membrane after exposure to 29°C.

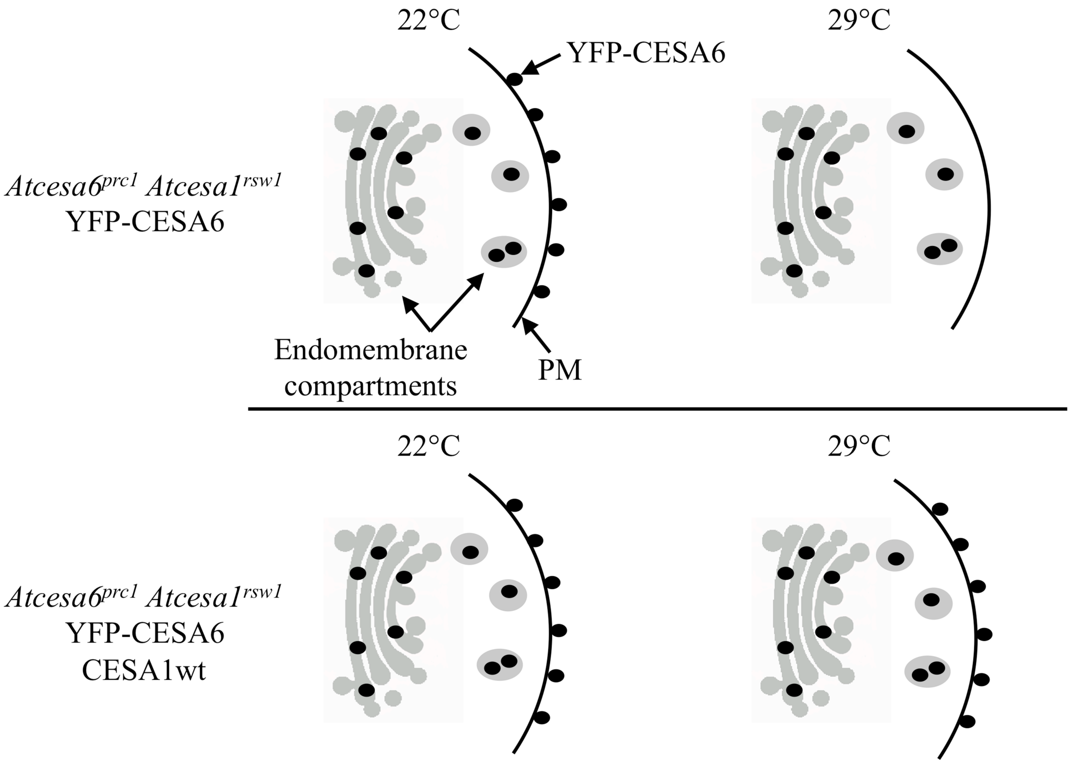

Table S1. Table of statistical information describing the computational modeling data.

		N-decoys	top cut	N-cluster	% of the top cut	RMSD-cut	Centroid ID	Centroid-REU	RMSD-Low	Best ID	REU-Low
AtCESA1	WT	24223	2422	477	0.197	6.23	10104	-41.14	15.76	21416	-79.80
AtCESA3	WT	20000	2000	279	0.140	6.00	3386	-28.13	16.25	13981	-81.44
AtCESA6	WT	17380	1738	362	0.208	6.25	11238	-24.39	13.72	12546	-86.56
AtCESA1	F954L	20000	2000	1161	0.581	6.00	16474	-41.79	16.74	19651	-72.97
AtCESA3	ixr1-2	23462	2346	229	0.098	6.05	4784	-28.72	13.04	5274	-76.08

Table S2. The results from a BLAST search of amino acids 820-890 from GhCESA1 (GenBank Accession P93155) were used to create a visual representation of the evolutionary conservation of the protein sequence of the TMH5-6 region from CESAs depicted in Figure 1C.

GI Number	Peptide sequence
>1706956	WVIGGVSAHLFAVFQGFLKMLAGIDTNFTVTAK-A-A-D-D-AD--FGELYIVKWTTLLIPPTTLLIVNMVGVVAGFS
>108767394	WVIGGVSAHLFAVFQGFLKMLAGIDTNFTVTAK-A-A-D-D-AD--FGELYIVKWTTLLIPPTTLLIVNMVGVVAGFS
>17226294	WVIGGVSAHLFAVFQGFLKMLAGIDTNFTVTAK-A-A-D-D-AD--FGELYIVKWTTLLIPPTTLLIVNMVGVVAGFS
>508780695	WVIGGVSAHLFAVFQGFLKMLAGIDTNFTVTAK-A-A-D-D-AD--FGELYIVKWTTLLIPPTTLLIVNMVGVVAGFS
>345104037	WVIGGVSAHLFAVFQGFLKMLAGIDTNFTVTAK-A-A-D-D-AD--FGELYIVKWTTLLIPPTTLLIVNMVGVVAGFS
>345104001	WVIGGVSAHLFAVFQGFLKMLAGIDTNFTVTAK-A-A-D-D-AD--FGELYIVKWTTLLIPPTTLLIVNMVGVVAGFS
>324984027	WVIGGVSAHLFAVFQGFLKMLAGIDTNFTVTAK-A-A-D-D-AD--FGELYIVKWTTLLIPPTTLLIVNMVGVVAGFS
>188509978	WVIGGVSAHLFAVFQGFLKMLAGIDTNFTVTAK-A-A-D-D-AD--FGELYIVKWTTLLIPPTTLLIVNMVGVVAGFS
>345104007	WVIGGVSAHLFAVFQGFLKMLAGIDTNFTVTAK-A-A-D-D-AD--FGELYIVKWTTLLIPPTTLLIVNMVGVVAGFS
>1706956	WVIGGVSAHLFAVFQGFLKMLAGIDTNFTVTAK-A-A-D-D-AD--FGELYIVKWTTLLIPPTTLLIVNMVGVVAGFS
>345104031	WVIGGVSAHLFAVFQGFLKMLAGIDTNFTVTAK-A-A-D-D-AD--FGELYIVKWTTLLIPPTTLLIVNMVGVVAGFS
>345104011	WVIGGVSAHLFAVFQGFLKMLAGIDTNFTVTAK-A-A-D-D-AD--FGELYIVKWTTLLIPPTTLLIVNMVGVVAGFS
>345104005	WVIGGVSAHLFAVFQGFLKMLAGIDTNFTVTAK-A-A-D-D-AD--FGELYIVKWTTLLIPPTTLLIVNMVGVVAGFS
>345103997	WVIGGVSAHLFAVFQGFLKMLAGIDTNFTVTAK-A-A-D-D-AD--FGELYIVKWTTLLIPPTTLLIVNMVGVVAGFS
>324984035	WVIGGVSAHLFAVFQGFLKMLAGIDTNFTVTAK-A-A-D-D-AD--FGELYIVKWTTLLIPPTTLLIVNMVGVVAGFS
>345103995	WVIGGVSAHLFAVFQGFLKMLAGIDTNFTVTAK-A-A-D-D-AD--FGELYIVKWTTLLIPPTTLLIVNMVGVVAGFS
>345104009	WVIGGVSAHLFAVFQGFLKMLAGIDTNFTVTAK-A-A-D-D-AD--FGELYIVKWTTLLIPPTTLLIVNMVGVVAGFS
>345103999	WVIGGVSAHLFAVFQGFLKMLAGIDTNFTVTAK-A-A-D-D-AD--FGELYIVKWTTLLIPPTTLLIVNMVGVVAGFS
>324984037	WVIGGVSAHLFAVFQGFLKMLAGIDTNFTVTAK-A-A-D-D-AD--FGELYIVKWTTLLIPPTTLLIVNMVGVVAGFS
>345104017	WVIGGVSAHLFAVFQGFLKMLAGIDTNFTVTAK-A-A-D-D-AD--FGELYIVKWTTLLIPPTTLLIVNMVGVVAGFS
>345104035	WVIGGVSAHLFAVFQGFLKMLAGIDTNFTVTAK-A-A-D-D-AD--FGELYIVKWTTLLIPPTTLLIVNMVGVVAGFS
>345104027	WVIGGVSAHLFAVFQGFLKMLAGIDTSFTVTAK-A-A-D-D-AD--FGELYIVKWTTLLIPPTTLLIVNMVGVVAGFS
>508780696	WVIGGVSAHLFAVFQGFLKMLAGIDTNFTVTAK-A-A-D-D-AD--FGELYIVKWTTLLIPPTTLLIVNMVGVVAGFS
>188509962	WVIGGVSAHLFAVFQGFLKMLAGIDTNFTVTAK-A-A-E-D-AE--FGELYIVKWTTLLIPPTTLLIVNMVGVVAGFS
>449465018	WVIGGVSAHLFAVFQGFLKMLAGIDTNFTVTAK-A-A-D-D-AE--FGELYMVKWTTLLIPPTTLIVVNMVGVVAGFS
>148841129	WVIGGVSAHLFAVFQGFLKMLAGIDTNFTVTAK-A-A-D-D-AD--FGELYIVKWTTLLIPPTTLLIVNMVGVVAGFS

>444436408	WVIGGVSAHLFAVFQGFLKMLAGLDTNFTVTAK-A-A-D-D-AE--FGELYMIKWTTLLIPPTTLLIVNMVGVVAGFS
>449500838	WVIGGVSAHLFAVFQGFLKMLAGIDTNFTVTAK-A-A-D-D-AE--FGELYMVKWTTLLIPPTTLIVVNMVGVVAGFS
>340343831	WVIGGVSAHLFAVFQGFLKMLAGLDTNFTVTAK-A-A-D-D-AE--FGELYMIKWTTLLIPPTTLLIVNMVGVVAGFS
>212960342	WVIGGVSAHLFAVFQGFLKMLAGVDTNFTVTAK-A-A-D-D-AE--FGELYIIKWTTVLIPPTTLIIVNMVGVVAGFS
>47078498	WVIGGVSAHLFAVFQGFLKMLAGIDTNFTVTAK-A-A-E-D-AE--FGELYMVKWTTLLIPPTTLLIINIVGVVAGFS
>479017124	WVIGGVSAHLFAVFQGFLKMLAGLDTNFTVTTK-A-A-D-D-AE--FGELYMIKWTTLLIPPTTLLIVNMVGVVAGFS
>429326424	WVIGGVSAHLFAVFQGFLKMLAGIDTNFTVTAK-A-A-D-D-TE--FGELYMVKWTTLLIPPTTLLIINIVGVVAGFS
>332356339	WVIGGVSAHLFAVFQGFLKMLAGIDTNFTVTAK-A-A-D-D-TE--FGELYMVKWTTLLIPPTTLLIINIVGVVAGFS
>54112376	WVIGGVSAHLFAVFQGFLKMLAGIDTNFTVTAK-A-A-D-D-TE--FGELYMVKWTTLLIPPTTLLIINIVGVVAGFS
>67003907	WVIGGVSAHLFAVFQGFLKMLAGLDTNFTVTTK-A-A-D-D-AE--FGELYMIKWTTLLIPPTTLLIVNMVGVVAGFS
>404325916	WVIGGVSAHLFAVFQGFLKMLAGLDTNFTVTTK-A-A-D-D-AE--FGELYMIKWTTLLIPPTTLLIVNMVGVVAGFS
>383081823	WVIGGVSAHLFAVFQGFLKMLAGLDTNFTVTTK-A-A-D-D-AE--FGELYMIKWTTLLIPPTTLLIVNMVGVVAGFS
>327397145	WVIGGVSAHLFAVFQGFLKMLAGLDTNFTVTTK-A-A-D-D-AE--FGELYMIKWTTLLIPPTTLLIVNMVGVVAGFS
>383081827	WVIGGVSAHLFAVFQGFLKMLAGLDTNFTVTTK-A-A-D-D-AE--FGELYMIKWTTLLIPPTTLLIVNMVGVVAGFS
>183211888	WVIGGVSAHLFAVFQGFLKMLAGVDTNFTVTTK-A-A-D-D-AE--FGELYIIKWTTVLIPPTTLIIVNMVGVVAGFS
>224114633	WVIGGVSAHLFAVFQGFLKLLAGIDTNFTVTAK-A-A-D-D-TE--FGELYMVKWTTLLIPPTTLLIINIVGVVAGFS
>462410311	WVIGGVSAHLFAVFQGFLKMLAGIDTNFTVTSK-q-A-E-D-AD--FGELYMIKWTTLLIPPTTLLIVNMVGIVAGFS
>429326452	WVIGGVSAHLFAVFQGFLKMLAGIDTNFTVTAK-A-A-E-D-TE--FGELYMVKWTTLLIPPTTLLIINIVGVVAGFS
>561032887	WVIGGVSAHLFAVFQGFLKMLAGVDTNFTVTAK-A-A-E-D-SE--FGELYLVKWTTLLIPPTTLIIVNLVGVVAGFS
>332356341	WVIGGVSAHLFAVFQGFLKMLAGIDTNFTVTAK-A-A-E-D-TE--FGELYMVKWTTLLIPPTTLLIINIVGVVAGFS
>224076940	WVIGGVSAHLFAVFQGFLKMLAGIDTNFTVTAK-A-A-E-D-TE--FGELYMVKWTTLLIPPTTLLIINIVGVVAGFS
>356515180	WVIGGVSAHLFAVFQGFLKMLAGVDTNFTVTAK-A-A-D-D-TE--FGDLYIIKWTTLLIPPTTLIIINMVGVVAGFS
>359483534	WVIGGVSAHLFAVFQGFLKMVAGLDTNFTVTAK-A-A-D-D-GE--FGELYMIKWTTLLIPPTTLLIINLVGVVAGFS
>357519009	WVIGGsSAHLFAVFQGFLKMLAGVDTNFTVTAK-A-A-E-D-TE--FGELYIIKWTTLLIPPTTLIIINMVGVVAGFS
>356528340	WVIGGVSAHLFAVFQGLLKMLAGVDTNFTVTAK-A-A-E-D-SE--FGELYLVKWTTLLIPPTTLIVVNMVGVVAGFS
>255562464	WVIGGVSAHLFAVFQGFLKMLAGIDTNFTVTAK-A-A-E-D-TD--FGELYIVKWTTVLIPPTSLIIINIVGVVAGFS
>502078355	WVIGGVSAHLFAVFQGFLKMLAGVDTNFTVTAK-A-A-D-D-AE--FGDLYIIKWTTLLIPPTSLIIINLVGVVAGFS
>502078352	WVIGGVSAHLFAVFQGFLKMLAGVDTNFTVTAK-A-A-D-D-AE--FGDLYIIKWTTLLIPPTSLIIINLVGVVAGFS
>557552148	WVIGGVSAHLFAVFQGFLKMLAGLDTNFTVTSK-A-A-D-D-lE--FGELYIIKWTTLLIPPTSLIIVNMVGVVAGFS
>297740530	WVIGGVSAHLFAVFQGFLKMVAGLDTNFTVTAK-A-A-D-D-GE--FGELYMIKWTTLLIPPTTLLIINLVGVVAGFS
>557115229	WVIGGVSAHLFAVFQGFLKMLAGLDTNFTVTSK-T-A-E-D-lE--FGELYIVKWTTLLIPPTTLLIVNLVGVVAGFS
>4539397	WVIGGVSAHLFAVFQGFLKMLAGLDTNFTVTSK-T-A-D-D-lE--FGELYIVKWTTLLIPPTSLLIINLVGVVAGFS
>297800186	WVIGGVSAHLFAVFQGFLKMLAGLDTNFTVTSK-T-A-D-D-lE--FGELYIVKWTTLLIPPTSLLIINLVGVVAGFS
>410942750	WVIGGVSAHLFAVFQGFLKMLAGIDTNFTVTTK-S-A-E-D-TE--FGELYLIKWTTLLIPPTTLLIVNMVGVVAGFS
>18415170	WVIGGVSAHLFAVFQGFLKMLAGLDTNFTVTSK-T-A-D-D-lE--FGELYIVKWTTLLIPPTSLLIINLVGVVAGFS
>12836997	WVIGGVSAHLFAVFQGFLKMLAGLDTNFTVTSK-T-A-D-D-lE--FGELYIVKWTTLLIPPTSLLIINLVGVVAGFS
>561010067	WVIGGVSAHLFAVFQGFLKMLAGVDTNFTVTAK-A-A-E-D-SE--FGELYMIKWTTLLIPPTTLIVINIVGVVAGFS
>241740158	WVIGGVSAHLFAVFQGFLKMLAGLDTNFTVTSK-T-A-E-D-lE--FGELYIVKWTTLLIPPTSLLIINLVGVVAGFS
>460374451	WVIGGVSAHLFAVFQGFLKMLAGIDTNFTVTAK-A-A-D-D-GE--FADLYLfKWTTVLIPPTTILIVNLVGVVAGFS
>241740153	WVIGGVSAHLFAVFQGFLKMLAGLDTNFTVTSK-T-A-E-D-lE--FGELYIVKWTTLLIPPTSLLVINLVGVVAGFS
>565441253	WVIGGVSAHLFAVFQGFLKMLAGLDTNFTVTSK-T-A-D-D-lE--FGELYIVKWTTLLIPPTSLLIINLVGVVAGFS

>548861573	WVIGGVSAHLFAVFQGLLKVLAGVDTNFTVTSK-A-A-E-D-SD--FGELYLfKWTTLLIPPTTLLIINMVGVVAGvS
>4115905	WVIGGVSAHLFAVFQGFLKMLAGIDTNFTVTAK-A-A-E-D-AE--FGELYMVKWTTLLIPPTTLLIINMsG-cAGFS
>565385016	WVIGGVSAHLFAVFQGLLKVLAGVDTNFTVTSK-A-A-D-D-AE--FGELYLfKWTTLLIPPTTLIILNMVGVVAGvS
>460404054	WVIGGVSAHLFAVFQGLLKVLAGVDTNFTVTSK-A-A-D-D-AE--FGELYLfKWTTLLIPPTTLIILNMVGVVAGvS
>508702453	WVIGGVSAHLFAVFQGLLKVLAGVDTNFTVTSK-A-A-D-D-AE--FGELYLfKWTTLLIPPTTLIILNMVGVVAGvS
>508702452	WVIGGVSAHLFAVFQGLLKVLAGVDTNFTVTSK-A-A-D-D-AE--FGELYLfKWTTLLIPPTTLIILNMVGVVAGvS
>561032284	WVIGGVSAHLFAVFQGLLKVLGGVDTNFTVTAK-A-A-D-D-AE--FGELYLfKWTTLLIPPTTLIILNMVGVVAGvS
>356528142	WVIGGVSAHLFAVFQGLLKVLGGVDTNFTVTAK-A-A-D-D-AE--FGELYLfKWTTLLIPPTTLIILNMVGVVAGvS
>255571220	WVIGGVSAHLFAVFQGLLKVLGGVDTNFTVTAK-A-A-D-D-AE--FGELYLfKWTTLLIPPTTLIILNMVGVVAGvS
>565384370	WVIGGVSAHLFAVFQGFLKMLAGIDTNFTVTTK-A-A-D-D-GE--FADLYLfKWTTVLIPPTTILIVNLVGVVAGFS
>527183369	WVIGGVSAHLFAVFQGFLKMLAGVDTNFTVTAK-A-A-D-D-TE--FGELYMIKWTTVLIPPTTILIVNMVGVVAGFS
>357449211	WVIGGVSAHLFAVFQGLLKVLAGVDTNFTVTAK-A-A-D-D-AE--FGELYLfKWTTLLIPPTTLIILNIVGVVAGvS
>391225927	WVIGGVSAHLFAVFQGLLKVLAGIDTNFTVTAK-G-A-E-D-GE--FGELYLfKWTTLLIPPTTLLVINLVGVVAGFS
>369762882	WVIGGVSAHLFAVFQGLLKVLAGVDTNFTVTAK-A-A-D-D-TE--FGELYLfKWTTLLIPPTTLIILNMVGVVAGvS
>33327261	WVIGGVSAHLFAVFQGLLKVLAGVDTNFTVTSK-S-A-D-D-AE--FGELYLfKWTTLLIPPTTLIILNMVGVVAGvS
>242054431	WVIGGVSAHLFAVFQGILKMIAGLDTNFTVTAK-A-T-D-D-AE--FGELYVfKWTTVLIPPTSILVLNMVGVVAGFS
>459958133	WVIGGVSAHLFAVFQGLLKVLAGVDTNFTVTSK-T-A-D-D-AE--FGELYLfKWTTLLIPPTTLIILNMVGVVAGvS
>183211894	WVIGGVSAHLFAVFQGLLKVLAGVDTNFTVTSK-T-A-D-D-AE--FGELYLfKWTTLLIPPTTLIILNMVGVVAGvS
>212960417	WVIGGVSAHLFAVFQGLLKVLAGVDTNFTVTSK-T-A-D-D-AE--FGELYLfKWTTLLIPPTTLIIMNMVGVVAGvS
>470107289	WVIGGVSAHLFAVFQGLLKVLAGVDTNFTVTSK-A-A-E-D-AE--FGELYLfKWTTLLIPPTTLIILNMVGVVAGvS
>67003909	WVIGGVSAHLFAVFQGLLKVLAGVDTNFTVTAK-A-A-E-D-SE--FGELYLfKWTTLLIPPTTLIILNMVGVVAGvS
>404325918	WVIGGVSAHLFAVFQGLLKVLAGVDTNFTVTAK-A-A-E-D-SE--FGELYLfKWTTLLIPPTTLIILNMVGVVAGvS
>340343833	WVIGGVSAHLFAVFQGLLKVLAGVDTNFTVTAK-A-A-E-D-SE--FGELYLfKWTTLLIPPTTLIILNMVGVVAGvS
>162955788	WVIGGVSAHLFAVFQGLLKVLAGVDTNFTVTAK-A-A-E-D-SE--FGELYLfKWTTLLIPPTTLIILNMVGVVAGvS
>162955782	WVIGGVSAHLFAVFQGLLKVLAGVDTNFTVTAK-A-A-E-D-SE--FGELYLfKWTTLLIPPTTLIILNMVGVVAGvS
>225438009	WVIGGVSAHLFAVFQGLLKVLAGVDTNFTVTSK-A-A-D-D-AE--FGDLYLfKWTTLLIPPTTLIILNMVGVVAGvS
>502085609	WVIGGVSAHLFAVFQGLLKVLAGVDTNFTVTAK-A-A-D-D-AE--FGDLYLfKWTTLLIPPTTLIILNIVGVVAGvS
>479017141	WVIGGVSAHLFAVFQGLLKVLAGVDTNFTVTAK-A-A-E-D-SE--FGELYLfKWTTLLIPPTTLIILNMVGVVAGvS
>297744201	WVIGGVSAHLFAVFQGLLKVLAGVDTNFTVTSK-A-A-D-D-AE--FGDLYLfKWTTLLIPPTTLIILNMVGVVAGvS
>212960378	WVIGGVSAHLFAVFQGLLKVLAGIDTNFTVTSK-A-T-D-D-eD--FGELYtfKWTTLLIPPTTILIINLVGVVAGIS
>183211892	WVIGGVSAHLFAVFQGLLKVLAGIDTNFTVTSK-A-T-D-D-eD--FGELYtfKWTTLLIPPTTILIINLVGVVAGIS
>369762884	WVIGGVSAHLFAVFQGLLKVLAGVDTNFTVTAK-A-A-E-D-TE--FGELYLfKWTTLLIPPTTLIILNMVGVVAGvS
>60299997	WVIGGVSAHFFAVFQGLLKVLAGIDTNFTVTAK-A-S-D-D-nE--FGELYafKWTTLLIPPTTLLVINLVGIVAGFS
>545721919	WVIGGVSAHLFAVFQGLLKVLGGVDTNFTVTSK-S-A-D-D-AE--FGELYLfKWTTLLIPPTTLIILNMVGVVAGvS
>545721917	WVIGGVSAHLFAVFQGLLKVLGGVDTNFTVTSK-S-A-D-D-AE--FGELYLfKWTTLLIPPTTLIILNMVGVVAGvS
>545721909	WVIGGVSAHLFAVFQGLLKVLGGVDTNFTVTSK-S-A-D-D-AE--FGELYLfKWTTLLIPPTTLIILNMVGVVAGvS
>545721907	WVIGGVSAHLFAVFQGLLKVLGGVDTNFTVTSK-S-A-D-D-AE--FGELYLfKWTTLLIPPTTLIILNMVGVVAGvS
>545721903	WVIGGVSAHLFAVFQGLLKVLGGVDTNFTVTSK-S-A-D-D-AE--FGELYLfKWTTLLIPPTTLIILNMVGVVAGvS
>545721893	WVIGGVSAHLFAVFQGLLKVLGGVDTNFTVTSK-S-A-D-D-AE--FGELYLfKWTTLLIPPTTLIILNMVGVVAGvS
>545721887	WVIGGVSAHLFAVFQGLLKVLGGVDTNFTVTSK-S-A-D-D-AE--FGELYLfKWTTLLIPPTTLIILNMVGVVAGvS
>545721885	WVIGGVSAHLFAVFQGLLKVLGGVDTNFTVTSK-S-A-D-D-AE--FGELYLfKWTTLLIPPTTLIILNMVGVVAGvS

>545721879	WVIGGVSAHLFAVFQGLLKVLGGVDTNFTVTSK-S-A-D-D-AE--FGELYLfKWTTLLIPPTTLIILNMVGVVAGvS
>545721877	WVIGGVSAHLFAVFQGLLKVLGGVDTNFTVTSK-S-A-D-D-AE--FGELYLfKWTTLLIPPTTLIILNMVGVVAGvS
>545721867	WVIGGVSAHLFAVFQGLLKVLGGVDTNFTVTSK-S-A-D-D-AE--FGELYLfKWTTLLIPPTTLIILNMVGVVAGvS
>545721865	WVIGGVSAHLFAVFQGLLKVLGGVDTNFTVTSK-S-A-D-D-AE--FGELYLfKWTTLLIPPTTLIILNMVGVVAGvS
>545721857	WVIGGVSAHLFAVFQGLLKVLGGVDTNFTVTSK-S-A-D-D-AE--FGELYLfKWTTLLIPPTTLIILNMVGVVAGvS
>545721853	WVIGGVSAHLFAVFQGLLKVLGGVDTNFTVTSK-S-A-D-D-AE--FGELYLfKWTTLLIPPTTLIILNMVGVVAGvS
>545721849	WVIGGVSAHLFAVFQGLLKVLGGVDTNFTVTSK-S-A-D-D-AE--FGELYLfKWTTLLIPPTTLIILNMVGVVAGvS
>545721847	WVIGGVSAHLFAVFQGLLKVLGGVDTNFTVTSK-S-A-D-D-AE--FGELYLfKWTTLLIPPTTLIILNMVGVVAGvS
>545721843	WVIGGVSAHLFAVFQGLLKVLGGVDTNFTVTSK-S-A-D-D-AE--FGELYLfKWTTLLIPPTTLIILNMVGVVAGvS
>332356343	WVIGGVSAHLFAVFQGLLKVLGGVDTNFTVTSK-S-A-D-D-AE--FGELYLfKWTTLLIPPTTLIILNMVGVVAGvS
>254680873	WVIGGVSAHLFAVFQGLLKVLGGVDTNFTVTSK-S-A-D-D-AE--FGELYLfKWTTLLIPPTTLIILNMVGVVAGvS
>224065557	WVIGGVSAHLFAVFQGLLKVLGGVDTNFTVTSK-S-A-D-D-AE--FGELYLfKWTTLLIPPTTLIILNMVGVVAGvS
>47078492	WVIGGVSAHLFAVFQGLLKVLGGVDTNFTVTSK-S-A-D-D-AE--FGELYLfKWTTLLIPPTTLIILNMVGVVAGvS
>328496823	WVIGGVSAHLFAVFQGLLQVLAGVDTNFTVTAK-A-A-E-D-SE--FGELYLfKWTTLLIPPTTLIILNMVGVVAGvS
>328496821	WVIGGVSAHLFAVFQGLLQVLAGVDTNFTVTAK-A-A-E-D-SE--FGELYLfKWTTLLIPPTTLIILNMVGVVAGvS
>514781896	WVIGGVSAHLFAVFQGILKMIAGLDTNFTVTAK-A-T-D-D-TE--FGELYVfKWTTVLIPPTTILVLNLVGVVAGFS
>3511285	WVIGGVSGHLFAVFQGFFKVLGGVDTNFTVTSK-S-A-D-D-AE--FGELYLfKWTTLLIPPTTLIILNMVGVVAGvS
>449445624	WVIGGVSAHLFAVFQGLLKVLGGVDTNFTVTAK-A-A-E-D-TE--FGELYLfKWTTLLIPPTTLIILNMVGVVAGIS
>561011936	WVIGGVSAHLFAVFQGLLKVLAGVDTNFTVTSK-A-A-E-D-AE--FGELYLfKWTTLLIPPTTLIILNIVGVVAGvS
>462406151	WVIGGVSAHLFAVFQGLLKVLAGVDTNFTVTSK-A-G-D-D-AD--FSELYafKWTTLLIPPTTLLIINLIGVVAGvS
>357136452	WVIGGVSAHLFAVFQGILKMViGLDTNFTVTAK-A-A-E-D-GD--FGELYVfKWTTVLIPPTTILVLNLVGVVAGFS
>414586402	WVIGGVSAHLFAVFQGILKMIAGLDTNFTVTAK-A-T-D-D-TE--FGELYLfKWTTVLIPPTSILVLNLVGVVAGFS
>162458651	WVIGGVSAHLFAVFQGILKMIAGLDTNFTVTAK-A-T-D-D-TE--FGELYLfKWTTVLIPPTSILVLNLVGVVAGFS
>241740113	WVIGGVSAHLFAVFQGLLKVLAGIDTNFTVTSK-A-S-DeD-GD--FAELYLfKWTTLLIPPTTLLIVNLVGVVAGFS
>414586403	WVIGGVSAHLFAVFQGILKMIAGLDTNFTVTAK-A-T-D-D-TE--FGELYLfKWTTVLIPPTSILVLNLVGVVAGFS
>1706958	WVIGGVSAHLFAVFQGLLKVLAGVDTNFTVTAK-A-A-D-D-TE--FGELYLfKWTTLLIPPTTLIILNMVGVVAGvS
>502150029	WVIGGVSAHLFAVIQGLLKVLAGIDTNFTVTSK-A-A-D-D-eE--FGELYtIKWTTLLIPPTTILIINIVGVVAGvS
>302754674	WVIGGVSAHLFAVFQGLLKVLAGIDTNFTVTSK-T-S-D-D-eE--FGELYafKWTTLLIPPTTLLVINMIGVVAGIS
>302804316	WVIGGVSAHLFAVFQGLLKVLAGIDTNFTVTSK-T-S-D-D-eE--FGELYafKWTTLLIPPTTLLVINMIGVVAGIS
>302768006	WVIGGVSSHLFAVFQGLLKVLAGIDTNFTVTTK-A-A-E-D-eD--FAELYtfKWTTLLIPPTTLLVINMVGVVAGLS
>172044097	WVIGGVSAHLFAVFQGILKMIAGLDTNFTVTAK-A-T-D-D-TE--FGELYVfKWTTVLIPPTSILVLNLVGVVAGFS
>115439945	WVIGGVSAHLFAVFQGILKMIAGLDTNFTVTAK-A-T-D-D-TE--FGELYVfKWTTVLIPPTSILVLNLVGVVAGFS
>557546026	WVIGGVSAHLFAVFQGLLKVLAGVDTNFTVTSK-S-A-E-D-eE--FGELYLfKWTTLLIPPTTLIILNMVGVVAGvS
>479017159	WVIGGVSAHLFAVvQGLLKVLAGIDTNFTVTSK-A-S-D-D-eD--FGELYafKWTTLLIPPTTILIINLVGVVAGIS
>479280309	WVIGGVSAHLFAVvQGLLKVLAGIDTNFTVTSK-A-S-D-D-eD--FGELYafKWTTLLIPPTTILIINLVGVVAGIS
>444436396	WVIGGVSAHLFAVvQGLLKVLAGIDTNFTVTSK-A-S-D-D-eD--FGELYafKWTTLLIPPTTILIINLVGVVAGIS
>67003911	WVIGGVSAHLFAVvQGLLKVLAGIDTNFTVTSK-A-S-D-D-eD--FGELYafKWTTLLIPPTTILIINLVGVVAGIS
>340343835	WVIGGVSAHLFAVvQGLLKVLAGIDTNFTVTSK-A-S-D-D-eD--FGELYafKWTTLLIPPTTILIINLVGVVAGIS
>302760255	WVIGGVSSHLFAVFQGLLKVLAGIDTNFTVTSK-A-T-D-D-eE--FGELYtLKWTTLLVPPTTLLIINLVGVVAGLA
>125572032	WVIGGVSAHLFAVFQGILKMIAGLDTNFTVTAK-A-T-D-D-TE--FGELYVfKWTTVLIPPTSILVLNLVGVVAGFS
>462395079	WVIGGVSAHFFAVFQGLLKVLfGVDTNFTVTSK-A-A-E-D-AE--FGELYLfKWTTLLIPPTTLIILNMVGVVAGIS

>462400201	WVIGGVSAHLFAVIQGLLKVLAGIDTNFTVTAK-S-S-D-D-eD--FGELYafKWTTLLIPPTTILVINLVGVVAGIS
>302753734	WVIGGVSSHLFAVFQGLLKVLAGIDTNFTVTTK-A-A-E-D-eD--FAELYtfKWTTLLIPPTTLIVINMVGVVAGLS
>356530215	WVIGGVSAHLFAVFQGLLKVLGGVDTNFTVTAK-A-A-E-D-TE--FGELYLfKWTTLLIPPTTLIILNIVGVVAGvS
>545721841	WVIGGVSAHLFAdFQGLLKVLGGVDTNFTVTSK-S-A-D-D-AE--FGELYLfKWTTLLIPPTTLIILNMVGVVAGvS
>356562551	WVIGGVSAHLFAVIQGLLKVLAGIDTNFTVTSK-A-A-D-D-eE--FGELYtfKWTTLLIPPTTILIINIVGVVAGIS
>356562549	WVIGGVSAHLFAVIQGLLKVLAGIDTNFTVTSK-A-A-D-D-eE--FGELYtfKWTTLLIPPTTILIINIVGVVAGIS
>356500681	WVIGGVSAHLFAVIQGLLKVLAGIDTNFTVTSK-A-A-D-D-eE--FGELYtfKWTTLLIPPTTILIINIVGVVAGIS
>548853484	WVIGGVSAHLFAVFQGLLKVLAGIDTNFTVTSK-A-G-D-D-SE--FSELYafKWTTLLIPPTTLLIINLIGVVAGIS
>302754590	WVIGGVSSHLFAVFQGLLKVLAGIDTNFTVTSK-S-A-D-D-eD--FGELYefKWTTLLIPPTTLIIVNLVGVAAGIS
>302804400	WVIGGVSSHLFAVFQGLLKVLAGIDTNFTVTSK-S-A-D-D-eD--FGELYefKWTTLLIPPTTLIIVNLVGVAAGIS
>460394041	WVIGGVSAHLFAVvQGLLKILAGIDTNFTVTSK-A-T-D-D-eD--FGELYafKWTTLLIPPTTILIINLVGVVAGIS
>565353400	WVIGGVSAHLFAVvQGLLKILAGIDTNFTVTSK-A-T-D-D-eD--FGELYafKWTTLLIPPTTILIINLVGVVAGIS
>357479993	WVIGGVSAHLFAVIQGLLKVLAGIDTNFTVTSK-A-T-D-D-eE--FGELYaIKWTTLLIPPTTILIINIVGVVAGIS
>386576418	WVIGGVSAHLFAVFQGFLKMLAGIDTNFTVTAK-A-A-D-D-GE--FGDLYIfKWTTVLIPPTTILILNLVGVVAGFS
>359484896	WVIGGVSAHLFAVvQGLLKVLAGIDTNFTVTSK-A-V-D-D-eE--FGELYtfKWTTLLIPPTTLLIINLVGVVAGIS
>225445816	WVIGGVSAHLFAVvQGLLKVLAGIDTNFTVTSK-A-V-D-D-eE--FGELYtfKWTTLLIPPTTLLIINLVGVVAGIS
>147778742	WVIGGVSAHLFAVvQGLLKVLAGIDTNFTVTSK-A-V-D-D-eE--FGELYtfKWTTLLIPPTTLLIINLVGVVAGIS
>407098206	WVIGGVSAHLFAVFQGLLKVLAGVDTNFTVTSK-G-G-D-D-AE--FAELYafKWTTLLIPPTTLLIINIVGVVAGIS
>550336663	WVIGGVSAHLFAVvQGLLKVLAGIDTNFTVTSK-A-T-D-D-dD--FGELYafKWTTLLIPPTTILIINLVGVVAGvS
>429326438	WVIGGVSAHLFAVvQGLLKVLAGIDTNFTVTSK-A-T-D-D-dD--FGELYafKWTTLLIPPTTILIINLVGVVAGvS
>429326422	WVIGGVSAHLFAVvQGLLKVLAGIDTNFTVTSK-A-T-D-D-dD--FGELYafKWTTLLIPPTTILIINLVGVVAGvS
>332356347	WVIGGVSAHLFAVvQGLLKVLAGIDTNFTVTSK-A-T-D-D-dD--FGELYafKWTTLLIPPTTILIINLVGVVAGvS
>332356345	WVIGGVSAHLFAVvQGLLKVLAGIDTNFTVTSK-A-T-D-D-dD--FGELYafKWTTLLIPPTTILIINLVGVVAGvS
>224143919	WVIGGVSAHLFAVvQGLLKVLAGIDTNFTVTSK-A-T-D-D-dD--FGELYafKWTTLLIPPTTILIINLVGVVAGvS
>224088330	WVIGGVSAHLFAVvQGLLKVLAGIDTNFTVTSK-A-T-D-D-dD--FGELYafKWTTLLIPPTTILIINLVGVVAGvS
>159885667	WVIGGVSAHLFAVvQGLLKVLAGIDTNFTVTSK-S-S-D-D-eD--FGELYafKWTTLLIPPTTILIINLVGVVAGIS
>470128264	WVIGGVSAHLFAVFQGLLKVLAGVDTNFTVTSK-G-G-D-D-AE--FSELYafKWTTLLIPPTTLLIINIVGVVAGIS
>401466650	WVIGGVSAHLFAVIQGLLKILAGIDTNFTVTSK-A-S-D-D-eE--FGELYafKWTTLLIPPTTILIINLVGVVAGIS
>168049043	WVIGGVSAHLFALFQGLLKVLAGIDTNFTVTSK-q-A-E-D-eD--FAELYMIKWTALLIPPTTLLVINMIGVVAGIS
>561006819	WVIGGVSAHLFAVFQGLLKVLAGIDTNFTVTSK-A-S-DeD-GD--FAELYMfKWTTLLIPPTTLLIINMVGVVAGIS
>168010390	WVIGGVSAHLFALFQGLLKVFAGVDTNFTVTSK-q-A-D-D-eD--FGELYMLKWTSLLIPPTTILILNLVGVVAGIS
>114793217	WVIGGVSAHLFALFQGLLKVFAGVDTNFTVTSK-q-A-D-D-eD--FGELYMLKWTSLLIPPTTILILNLVGVVAGIS
>548831070	WVIGGVSAHLFAVvQGLLKVLAGIDTNFTVTSK-A-T-D-D-eE--FGELYafKWTTLLIPPTTLLIINLVGVVAGvS
>225457723	WVIGGVSAHLFAVFQGLLKVLAGVDTNFTVTSK-A-G-D-D-VE--FSELYafKWTTLLIPPTTLLIINLIGVVAGIS
>297810629	WVIGGVSAHLFAVFQGILKVLAGIDTNFTVTSK-A-S-DeD-GD--FAELYLfKWTTLLIPPTTLLIVNLVGVVAGvS
>212960446	WVIGGVSAHLFAVFQGLLKVLAGVDTNFTVTSK-A-G-D-D-Aa--FSELYafKWTTLLIPPTTLLIINLIGVVAGvS
>557101369	WVIGGISAHLFAVvQGLLKILAGIDTNFTVTSK-A-T-D-D-dD--FGELYafKWTTLLIPPTTVLIINIVGVVAGIS
>565457947	WVIGGISAHLFAVvQGLLKILAGIDTNFTVTSK-A-T-D-D-dD--FGELYafKWTTLLIPPTTVLIINIVGVVAGIS
>4886756	WVIGGISAHLFAVvQGLLKILAGIDTNFTVTSK-A-T-D-D-dD--FGELYafKWTTLLIPPTTVLIINIVGVVAGIS
>241740147	WVIGGISAHLFAVvQGLLKILAGIDTNFTVTSK-A-T-D-D-dD--FGELYafKWTTLLIPPTTVLIINIVGVVAGIS
>297811887	WVIGGISAHLFAVvQGLLKILAGIDTNFTVTSK-A-T-D-D-dD--FGELYafKWTTLLIPPTTVLIINIVGVVAGIS

>168010279	WVIGGVSAHLFAVFQGLLKVFAGIDTNFTVTSK-S-S-E-D-eD--FGELYafKWTSLLIPPTTLLIINLVGVVAGIS
>15237958	WVIGGISAHLFAVvQGLLKILAGIDTNFTVTSK-A-T-D-D-dD--FGELYafKWTTLLIPPTTVLIINIVGVVAGIS
>326514590	WVIGGVSAHLFAVFQGILKMViGLDTNFTVTSK-A-A-E-D-GD--FAELYVfKWTTVLIPPTTILVLNLVGVVAGFS
>15238454	WVIGGVSAHLFAVFQGILKVLAGIDTNFTVTSK-A-S-DeD-GD--FAELYLfKWTTLLIPPTTLLIVNLVGVVAGvS
>385718957	WVIGGVSAHLFAVFQGLLKVLAGVDTNFTVTSK-G-G-D-D-AE--FSELYafKWTTLLIPPTTLLIINLIGVVAGvS
>527188907	WVIGGISAHLFAVvQGLLKIIAGIDTNFTVTSK-A-S-D-D-eD--FSELYtfKWTTLLIPPTTILIINMVGVVAGIS
>2827143	WVIGGVSAHLFAVFQGILKVLAGIDTNFTVTSK-A-S-DeD-GD--FAELYLfKWTTLLIPPTTLLIVNLVGVVAGvS
>561011482	WVIGGVSAHLFAVIQGLLKVLAGIDTNFTVTSK-A-T-D-D-eE--FGELYtfKWTTLLIPPTTILIINIVGVVAGIS
>356517040	WVIGGVSAHLFAVIQGLLKVLAGIDTNFTVTSK-A-T-D-D-eE--FGELYtfKWTTLLIPPTTILIINIVGVVAGIS
>356508362	WVIGGVSAHLFAVIQGLLKVLAGIDTNFTVTSK-A-T-D-D-eE--FGELYtfKWTTLLIPPTTILIINIVGVVAGIS
>470101367	WVIGGVSAHLFAVIQGLLKVLAGIDTSFTVTAK-S-S-D-D-eD--FGELYafKWTTLLIPPTTILVINLVGVVAGIS
>460367538	WVIGGVSAHLFAVFQGLLKVLAGIDTNFTVTSK-A-S-DeD-GD--FAELYMfKWTTLLIPPTTLLIVNLVGVVAGIS
>565357228	WVIGGVSAHLFAVFQGLLKVLAGIDTNFTVTSK-A-S-DeD-GD--FAELYLfKWTTLLIPPTTLLIVNLVGVVAGIS
>356530659	WVIGGVSAHLFAVFQGLLKVLAGIDTNFTVTSK-A-S-DeD-GD--FAELYMfKWTTLLIPPTTLLIINLVGVVAGIS
>508704786	WVIGGVSSHLFAVFQGLLKVLAGIDTNFTVTSK-A-S-DeD-GD--FAELYLfKWTTLLIPPTTLLIINLVGVVAGIS
>376315428	WVIGGISAHLFAVIQGLLKVLAGIDTNFTVTSK-A-T-D-D-eE--FGELYtfKWTTLLIPPTTVLIINLVGVVAGIS
>356557164	WVIGGVSAHLFAVFQGLLKVLAGIDTNFTVTSK-A-S-DeD-GD--FAELYLfKWTTLLIPPTTLLIINLVGVVAGIS
>291002735	WVIGGISAHLFAVIQGLLKVLAGIDTNFTVTSK-A-T-D-D-eE--FGELYtfKWTTLLIPPTTVLIINLVGVVAGIS
>340343837	WVIGGVSAHLFAVFQGLLKVLAGIDTNFTVTSK-A-S-DeD-GD--FAELYMfKWTTLLIPPTTLLIINLVGVVAGIS
>356548925	WVIGGVSAHLFAVFQGLLKVLAGIDTNFTVTSK-A-S-DeD-GD--FAELYMfKWTTLLIPPTTLLIINMIGVVAGIS
>548840773	WVIGGVSAHLFAVFQGLLKVLAGIDTNFTVTSK-A-T-DeD-GD--FAELYMfKWTTLLIPPTTLLIINLVGVVAGIS
>225450119	WVIGGVSAHLFAVFQGLLKVLAGIDTNFTVTSK-A-S-DeD-GD--FAELYMfKWTTLLIPPTTLLIINLVGVVAGIS
>449470291	WVIGGISAHLFAVIQGLLKVLAGIDTSFTVTSK-A-T-D-D-eD--FGELYafKWTTLLIPPTTILIINLVGVVAGIS
>33186651	WVIGGVSAHLFAVFQGLLKVLAGIDTNFTVTSK-A-T-DeD-GD--FAELYLfKWTTLLIPPTTLLIVNLVGVVAGIS
>168029238	WVIGGVSAHLFALFQGLLKVFAGIDTNFTVTSK-q-A-E-D-eD--FAELYMIKWTALLIPPTTLLVINMIGVVAGIS
>299109313	WVIGGVSAHLFAVFQGILKMViGLDTNFTVTSK-A-A-E-D-GD--FAELYVfKWTTVLIPPTTILVLNLVGVVAGFS
>312281585	WVIGGVSAHLFAVFQGvLKVLAGVDTNFTVTSK-A-S-DeD-GD--FAELYLfKWTTLLIPPTTLLIVNLVGVVAGvS
>325464701	WVIGGVSAHLFAVFQGLLKVLAGIDTNFTVTSK-A-S-DeD-GD--FAELYMfKWTTLLIPPTTLLIINLVGVVAGIS
>347953829	WVIGGVSAHLFAVFQGLLKVLAGIDTNFTVTSK-A-S-DeD-GD--FAELYMfKWTTLLIPPTTLLIINLVGVVAGIS
>347953823	WVIGGVSAHLFAVFQGLLKVLAGIDTNFTVTSK-A-S-DeD-GD--FAELYMfKWTTLLIPPTTLLIINLVGVVAGIS
>557100069	WVIGGVSAHLFAVFQGvLKVLAGVDTNFTVTSK-A-S-DeD-GD--FAELYLfKWTTLLIPPTTLLIVNLVGVVAGvS
>347953861	WVIGGVSAHLFAVFQGLLKVLAGIDTNFTVTSK-A-S-DeD-GD--FAELYMfKWTTLLIPPTTLLIINLVGVVAGIS
>347953853	WVIGGVSAHLFAVFQGLLKVLAGIDTNFTVTSK-A-S-DeD-GD--FAELYMfKWTTLLIPPTTLLIINLVGVVAGIS
>347953825	WVIGGVSAHLFAVFQGLLKVLAGIDTNFTVTSK-A-S-DeD-GD--FAELYMfKWTTLLIPPTTLLIINLVGVVAGIS
>565457903	WVIGGVSAHLFALFQGLLKVLAGVDTNFTVTSK-A-A-D-D-GE--FSELYIfKWTSLLIPPTTLLIINVIGVIVGIS
>347953863	WVIGGVSAHLFAVFQGLLKVLAGIDTNFTVTSK-A-S-DeD-GD--FAELYMfKWTTLLIPPTTLLIINLVGVVAGIS
>449436301	WVIGGVSAHLFAVFQGLLKVLAGIDTNFTVTSK-A-S-DeD-GD--YAELYMfKWTTLLIPPTTLLIVNLVGVVAGIS
>561033214	WVIGGVSSHLFALFQGLLKVLAGVNTNFTVTSK-A-A-D-D-GD--FSELYIfKWTSLLIPPTTLLIINIVGVVVGIS
>33413768	WVIGGVSAHLFAVFQGLLKVLAGVDTNFTVTAK-A-A-E-D-TE--FGELYLLKWTTLLIPPTTLIILNMVGVVAGvS
>347953867	WVIGGVSAHLFAVFQGLLKVLAGIDTNFTVTSK-A-S-DeD-GD--FAELYMfKWTTLLIPPTTLLIINLVGVVAGIS
>347953857	WVIGGVSAHLFAVFQGLLKVLAGIDTNFTVTSK-A-S-DeD-GD--FAELYMfKWTTLLIPPTTLLIINLVGVVAGIS

>347953855	WVIGGVSAHLFAVFQGLLKVLAGIDTNFTVTSK-A-S-DeD-GD--FAELYMfKWTTLLIPPTTLLIINLVGVVAGIS
>347953827	WVIGGVSAHLFAVFQGLLKVLAGIDTNFTVTSK-A-S-DeD-GD--FAELYMfKWTTLLIPPTTLLIINLVGVVAGIS
>325464707	WVIGGVSAHLFAVFQGLLKVLAGIDTNFTVTSK-A-S-DeD-GD--FAELYMfKWTTLLIPPTTLLIINLVGVVAGIS
>508717391	WVIGGVSAHLFAVFQGLLKVLAGIDTNFTVTSK-A-S-DeD-GD--FAELYLfKWTTLLIPPTTLLIINLVGVVAGvS
>6446577	WVIGGVSAHLFAVFQGLLKVLAGIDTNFTVTSK-A-S-DeD-GD--FAELYMfKWTTLLIPPTTLLIINLVGVVAGIS
>347953851	WVIGGVSAHLFAVFQGLLKVLAGIDTNFTVTSK-A-S-DeD-GD--FAELYMfKWTTLLIPPTTLLIINLVGVVAGIS
>347953843	WVIGGVSAHLFAVFQGLLKVLAGIDTNFTVTSK-A-S-DeD-GD--FAELYMfKWTTLLIPPTTLLIINLVGVVAGIS
>347953835	WVIGGVSAHLFAVFQGLLKVLAGIDTNFTVTSK-A-S-DeD-GD--FAELYMfKWTTLLIPPTTLLIINLVGVVAGIS
>347953831	WVIGGVSAHLFAVFQGLLKVLAGIDTNFTVTSK-A-S-DeD-GD--FAELYMfKWTTLLIPPTTLLIINLVGVVAGIS
>347953821	WVIGGVSAHLFAVFQGLLKVLAGIDTNFTVTSK-A-S-DeD-GD--FAELYMfKWTTLLIPPTTLLIINLVGVVAGIS
>325464703	WVIGGVSAHLFAVFQGLLKVLAGIDTNFTVTSK-A-S-DeD-GD--FAELYMfKWTTLLIPPTTLLIINLVGVVAGIS
>225428372	WVIGGVSAHLFAVFQGLLKVLAGIDTDFTVTSK-A-G-D-D-eD--FSELYafKWTTLLIPPTTLLIINLIGVVAGvS
>254554078	WVIGGISAHLFAVvQGLLKILAGIDTNFTVTSK-A-T-D-D-eE--FGELYtfKWTTLLIPPTTVLVINLVGVVAGIS
>224138030	WVIGGVSAHLFAVFQGLLKVLAGIDTNFTVTSK-A-S-DeD-GD--FtELYMfKWTTLLIPPTTLLIINLVGVVAGvS
>224090220	WVIGGVSAHLFAVFQGLLKVLAGIDTNFTVTSK-A-S-DeD-GD--FtELYMfKWTTLLIPPTTLLIINLVGVVAGvS
>168001214	WVIGGVSAHLFALFQGLLKVFAGIDTNFTVTSK-T-G-E-D-eD--FGELYaLKWTSLLIPPTTLLIfNMVGVVAGIS
>508784222	WVIGGISAHLFAVvQGLLKVLAGIDTNFTVTSK-A-T-D-D-eE--FGELYafKWTTLLIPPTTVLIINLVGVVAGvS
>449503806	WVIGGVSAHLFAVFQGLLKVLAGIDTNFTVTSK-A-S-DeD-GD--FAELYMfKWTTLLIPPTTLLIINIVGVVAGIS
>550320901	WVIGGVSAHLFAVFQGLLKVLAGIDTNFTVTSK-A-S-DeD-GD--FtELYMfKWTTLLIPPTTLLIINLVGVVAGvS
>429326450	WVIGGVSAHLFAVFQGLLKVLAGIDTNFTVTSK-A-S-DeD-GD--FtELYMfKWTTLLIPPTTLLIINLVGVVAGvS
>347953839	WVIGGVSAHLFAVFQGLLKVLAGIDTNFTVTSK-A-S-DeD-GD--FAELYMfKWTTLLIPPTTLLIINLVGVVAGIS
>356544169	WVIGGVSAHLFAVFQGLLKVLAGIDTNFTVTSK-A-S-DeD-GD--FAELYMfKWTTLLIPPTTLLIINLVGVVAGIS
>561022144	WVIGGVSAHLFAVFQGLLKVLAGIDTNFTVTSK-A-T-DeD-GD--FAELYMfKWTTLLIPPTTLLIINLVGVVAGIS
>449463334	WVIGGVSAHLFAVFQGLLKVLAGIDTNFTVTSK-A-S-DeD-GD--FAELYMfKWTTLLIPPTTLLIINIVGVVAGIS
>255585040	WVIGGISAHLFAVvQGLLKVLAGIDTNFTVTSK-A-T-D-D-eD--FAELYafKWTTLLIPPTTILIINLVGVVAGvS
>23534479	WVIGGVSAHLFAVvQGLLKVLAGIDlNFTVTSK-A-T-D-D-dD--FGELYafKWTTLLIPPTTILIINLVGVVAGvS
>550324734	WVIGGVSAHLFAVFQGLLKVLAGVDTNFTVTSK-G-G-D-D-dE--FSELYafKWTTLLIPPTTLLIINLVGVVAGvS
>429326430	WVIGGVSAHLFAVFQGLLKVLAGVDTNFTVTSK-G-G-D-D-dE--FSELYafKWTTLLIPPTTLLIINLVGVVAGvS
>319659269	WVIGGVSAHLFAVFQGLLKVLAGVDTNFTVTSK-G-G-D-D-dE--FSELYafKWTTLLIPPTTLLIINLVGVVAGvS
>313671704	WVIGGVSAHLFAVFQGLLKVLAGVDTNFTVTSK-G-G-D-D-dE--FSELYafKWTTLLIPPTTLLIINLVGVVAGvS
>224123782	WVIGGVSAHLFAVFQGLLKVLAGVDTNFTVTSK-G-G-D-D-dE--FSELYafKWTTLLIPPTTLLIINLVGVVAGvS
>224123130	WVIGGVSAHLFAVFQGLLKVLAGVDTNFTVTSK-G-G-D-D-dE--FSELYafKWTTLLIPPTTLLIINLVGVVAGvS
>37781495	WVIGGVSAHLFAVFQGLLKVLAGVDTNFTVTSK-G-G-D-D-dE--FSELYafKWTTLLIPPTTLLIINLVGVVAGvS
>475595757	WVIGGVSAHLFAVFQGILKMViGLDTNFTVTSK-A-A-E-D-GD--FAELYVfKWTTVLIPPTTILVLNLVGVVAGFS
>502127947	WVIGGVSAHLFAVFQGLLKVLAGIDTNFTVTSK-A-S-DeD-GD--FqELYMfKWTTLLIPPTTLLIINLVGVVAGIS
>67003917	WVIGGVSAHLFAVFQGLLKVLAGVDTNFTVTSK-G-G-D-D-kE--FSELYafKWTTLLIPPTTLLIINLIGVVAGvS
>49615365	WVIGGVSAHLFAVvQGLLKVLAGIDTNFTVTSK-A-S-DeD-GD--FAELYMfKWTTLLIPPTTLLIINMVGVVAGIS
>508704787	WVIGGVSSHLFAVFQGLLKVLAGIDTNFTVTSK-A-S-DeD-GD--FAELYLfKWTTLLIPPTTLLIINLVGVVAGIS
>73810227	WVIGGVSAHLFALFQGLLKVFAGIDTNFTVTSK-q-A-E-D-eD--FAELYMIKWTALLIPPTTLIVINMIGVVAGIS
>73810225	WVIGGVSAHLFALFQGLLKVFAGIDTNFTVTSK-q-A-E-D-eD--FAELYMIKWTALLIPPTTLIVINMIGVVAGIS
>114793219	WVIGGVSAHLFALFQGLLKVFAGIDTNFTVTSK-q-A-E-D-eD--FAELYMIKWTALLIPPTTLIVINMIGVVAGIS

>114793221	WVIGGVSAHLFALFQGLLKVFAGIDTNFTVTSK-q-A-E-D-eD--FAELYMIKWTALLIPPTTLIVINMIGVVAGIS
>508704788	WVIGGVSSHLFAVFQGLLKVLAGIDTNFTVTSK-A-S-DeD-GD--FAELYLfKWTTLLIPPTTLLIINLVGVVAGIS
>442736190	WVIGGVSAHLFAVFQGILKVLAGIDTNFTViSK-A-S-DeD-GD--FAELYLfKWTTLLIPPTTLLIVNLVGVVAGvS
>48995368	WVIGGVSAHLFAVFQGLLKVFAGIDTNFTVTSK-S-S-E-D-eD--FGELYafKWTSLLIPPTTLLIINLVGVVAGIS
>508784205	WVIGGTSAHLFAVFQGLLKVLAGIDTNFTVTSK-A-SdD-D-GD--FAELYVfKWTTLLIPPTTVLIVNLVGIVAGvS
>442736191	WVIGGVSAHLFAVFQGILKVLAGIDTNFTViSK-A-S-DeD-GD--FAELYLfKWTTLLIPPTTLLIVNLVGVVAGvS
>376315430	WVIGGVSAHLFAVFQGLLKVLAGIDTNFTVTSK-A-S-DeD-GD--FAELYMfKWTTLLIPPTTLLIINLVGVVAGvS
>242047858	WVIGGISAHLFAVFQGLLKVLAGIDTNFTVTSK-A-S-DeD-GD--FAELYMfKWTTLLIPPTTILIINLVGVVAGIS
>224106083	WVIGGVSAHLFAVFQGLLKVLAGIDTNFTVTSK-S-S-DeD-GD--FtELYMfKWTTLLIPPTTLLIVNLVGVVAGIS
>429326434	WVIGGVSAHLFAVFQGLLKVLAGIDTNFTVTSK-S-S-DeD-GD--FtELYMfKWTTLLIPPTTLLIVNLVGVVAGIS
>475553219	WVIGGVSAHLFAVFQGFLKVLGGVDTNFTVTSK-AgA-D-E-ADa-FGDLYLfKWTTLLIPPTTLIIINMVGIVAGvS
>357146541	WVIGGVSAHLFAVFQGFLKVLGGVDTNFTVTSK-A-AgD-E-ADa-FGDLYLfKWTTLLIPPTTLIIINMVGIVAGvS
>332356349	WVIGGVSAHLFAVFQGLLKVLAGIDTNFTVTSK-S-S-DeD-GD--FtELYMfKWTTLLIPPTTLLIVNLVGVVAGIS
>298716928	WVIGGVSAHLFAVFQGFLKVLGGVDTNFTVTSK-AgA-D-E-ADa-FGDLYLfKWTTLLIPPTTLIIINMVGIVAGvS
>255582781	WVIGGVSAHLFAVFQGLLKVLAGIDTNFTVTSK-A-S-DeD-GD--FAELYMfKWTTLLVPPTTLLIINFIGVVAGIS
>162460995	WVIGGISAHLFAVFQGLLKVLAGIDTNFTVTSK-A-S-DeD-GD--FAELYMfKWTTLLIPPTTILIINLVGVVAGIS
>414588935	WVIGGISAHLFAVFQGLLKVLAGIDTNFTVTSK-A-S-DeD-GD--FAELYMfKWTTLLIPPTTILIINLVGVVAGIS
>125557649	WVIGGISAHLFAVFQGLLKVLAGIDTNFTVTSK-A-S-DeD-GD--FAELYMfKWTTLLIPPTTILIINLVGVVAGIS
>414883975	WVIGGISAHLFAVFQGLLKVLAGIDTNFTVTSK-A-S-DeD-GD--FAELYMfKWTTLLIPPTTILIINLVGVVAGIS
>297807011	WVIGGVSAHLFALFQGLLKVLAGVDTNFTVTSK-A-A-D-D-GE--FSDLYIfKWTSLLIPPTTLLIINVIGVIVGIS
>115471127	WVIGGISAHLFAVFQGLLKVLAGIDTNFTVTSK-A-S-DeD-GD--FAELYMfKWTTLLIPPTTILIINLVGVVAGIS
>376315426	WVIGGISAHLFAVvQGLLKVLAGIDTNFTVTSK-T-T-D-D-eE--FGELYtfKWTTLLIPPTTVLIINLVGVVAGIS
>162461169	WVIGGISAHLFAVFQGLLKVLAGIDTNFTVTSK-A-S-DeD-GD--FAELYMfKWTTLLIPPTTILIINLVGVVAGIS
>561028222	WVIGGVSAHLFAVIQGLLKVLAGIDTNFTVTSK-A-T-D-D-eE--FSELYtfKWTTLLIPPTTILIINIVGVVAGIS
>565462856	WVIGGVSAHLFAVvQGLLKVLAGIDTNFTVTSK-A-S-DeD-GD--FAELYLfKWTTLLIPPTTLLIVNLVGVVAGvS
>15242540	WVIGGVSAHLFALFQGLLKVLAGVETNFTVTSK-A-A-D-D-GE--FSELYIfKWTSLLIPPTTLLIINVIGVIVGIS
>341752658	WVIGGVSAHLFALFQGLLKVLAGVETNFTVTSK-A-A-D-D-GE--FSELYIfKWTSLLIPPTTLLIINVIGVIVGIS
>325464699	WVIGGVSAHLFTVFQGLLKVLAGIDTNFTVTSK-A-S-DeD-GD--FAELYMfKWTTLLIPPTTLLIINLVGVVAGIS
>307557871	WVIGGVSSHLFAVvQGLLKVLAGIDTNFTVTSK-A-S-DeD-GD--FAELYMfKWTTLLIPPTTLLIVNMVGVVAGIS
>557541309	WVIGGVSSHLFAVFQGLLKVLAGIDTNFTVTSK-A-S-DeD-GD--FtELYMfKWTTLLIPPTTLLVINLVGVVAGvS
>33413764	WVIGGVSAHLFAVFQGLLKVLAGVDTNFTVTAK-A-A-E-D-TE--FGELYLfKWTTLLIPPTTLIILNMVGVVAGvS
>565477904	WVIGGVSAHLFAVFQGLLKVLAGIDTNFTVTSK-A-S-DeD-GD--sAELYLIKWTTLLVPPTTLLIINLVGVVAGvS
>33413770	WVIGGVSAHLFAVFQGLLKVLAGVDTNFTVTAK-A-A-E-D-TE--FGELYLfKWTTLLIPPTTLIILNMVGVVAGvS
>224055195	WVIGGVSAHLFAVFQGLLKVLAGIDTNFTVTSK-A-S-DeD-Gg--FAELYLfKWTTLLIPPTTLLIVNLVGVVAGIS
>33413766	WVIGGVSAHLFAVFQGLLKVLAGVDTNFTVTAK-A-A-E-D-TE--FGELYLfKWTTLLIPPTTLIILNMVGVVAGvS
>48995376	WVIGGVSAHLFALFQGLLKVFAGVDTNFTVTSK-q-A-D-D-eD--FGELYMLKWTSLLIPPTTILILNLVGVVAGIS
>462416750	WVIGGVSAHLFAVvQGLLKVLAGIDTNFTVTSK-A-S-DeD-GD--FAELYMfKWTTLLIPPTTLLIINLVGVVAGIS
>449437126	WVIGGTSAHLFAVFQGLLKVLAGIDTNFTVTSK-A-S-DeD-GD--FAELYVfKWTSLLIPPTTVLIMNMVGIVAGvS
>385718955	WVIGGVSAHLFAVFQGLLKVLAGIDTNFTVTSK-A-G-DeE-GD--FtELYMfKWTTLLIPPTTLLIINLVGVVAGIS
>307557873	WVIGGVSSHLFAVFQGLLKVLAGIDTNFTVTSK-A-S-DeD-GD--sAELYMfKWTTLLIPPTTLLIVNMVGVVAGIS
>541135559	WVIGGTSAHLFAVFQGLLKVLAGIDTNFTVTSK-A-S-DeD-GD--FAELYVfKWTSLLIPPTTVLIVNLVGIVAGvS

>557553695	WVIGGTSAHLFAVFQGLLKVLAGIDTNFTVTSK-A-SdD-D-GD--FAELYVfKWTSLLIPPTTVLIVNLVGIVAGvS
>470110392	WVIGGTSAHLFAVFQGLLKVLAGIDTNFTVTSK-A-S-DeD-GD--FAELYVfKWTSLLIPPTTVLIVNLVGIVAGvS
>356507742	WVIGGTSAHLFAVFQGLLKVLAGIDTNFTVTSK-A-S-DeD-GD--FAELYVfKWTSLLIPPTTVLIVNLVGIVAGvS
>470141636	WVIGGVSAHLFAVvQGLLKVLAGIDTNFTVTSK-A-S-DeD-GD--FAELYMfKWTTLLIPPTTLLIINLVGVVAGIS
>241740141	WVIGGVSAHLFALFQGLLKVLAGVDTNFTVTSK-A-A-D-D-GE--FSDLYLfKWTSLLIPPTTLLIINVIGIVVGIS
>527203331	WVIGGVSSHLFAVvQGLLKVLAGIDTNFTVTSK-A-S-D-E-GD--FAELYMfKWTTLLVPPTTLLVINLVGVVAGIS
>114793226	WVIGGVSAHLFALFQGLLKVLAGIDTNFTVTSK-q-A-E-D-eD--FAELYMIKWTALLIPPTTLLVINMIGVVAGIS
>39933010	WVIGGVSAHLFAVFQGLLKVLAGIDTNFTVTSK-A-S-DeD-GD--sAELYLfKWTTLLIPPTTLLIVNLVGVVAGIS
>462418519	WIIGGTSAHLFAVFQGLLKVLAGIDTNFTVTSK-A-S-DeD-GD--FAELYVfKWTSLLIPPTTVLLVNMVGIVAGvS
>410825315	WVIGGVSAHLFAVFQGLLKVLGGVDTNFTVTSK-S-A-D-D-AE--FGELYLfKWTTLLIPPTTLIILNMVGVVAGvS
>429326428	WVIGGVSAHLFAVFQGLLKVLAGIDTNFTVTSK-A-S-DeD-GD--sAELYLfKWTTLLIPPTTLLIVNLVGVVAGIS
>242039429	WVIGGVSAHLFAVFQGFLKVLGGVDTSFTVTSK-A-AgD-E-ADa-FGELYLfKWTTLLVPPTTLIIINMVGIVAGvS
>297821377	WVIGGVSSHLFALFQGLLKVLAGVNTNFTVTSK-A-A-D-D-GE--FSELYIfKWTSLLIPPTTLLIINIVGVIVGvS
>410825302	WVIGGVSAHLFAVFQGLLKVLGGVDTNFTVTSK-S-A-D-D-AE--FGELYLfKWTTLLIPPTTLIILNMVGVVAGvS
>414876095	WVIGGTSAHLFAVFQGLLKVLAGIDTNFTVTSK-A-TdD-D-GD--FAELYVfKWTTLLIPPTTVLVINLVGIVAGvS
>414876094	WVIGGTSAHLFAVFQGLLKVLAGIDTNFTVTSK-A-TdD-D-GD--FAELYVfKWTTLLIPPTTVLVINLVGIVAGvS
>356515456	WVIGGTSAHLFAVFQGLLKVLAGIDTNFTVTSK-A-S-DeD-GD--FAELYVfKWTSLLIPPTTVLIVNLVGIVAGvS
>565430708	WVIGGVSAHLFALFQGLLKVLAGVDTNFTVTSK-A-A-D-D-GE--FSDLYLfKWTSLLIPPTTLLIINVIGVIVGvS
>297794069	WVIGGVSAHLFALFQGLLKVLAGVDTNFTVTSK-A-A-D-D-GE--FSDLYLfKWTSLLIPPTTLLIINVIGVIVGvS
>162955786	WVIGGVSAHLFAVFQGLLKVLAGIDTNFTVTSK-A-S-DeD-GD--sAELYMfKWTTLLIPPTTLLIINLVGVVAGIS
>410825309	WVIGGVSAHLFAVFQGLLKVLGGVDTNFTVTSK-S-A-D-D-AE--FGELYLfKWTTLLIPPTTLIILNMVGVVAGvS
>67003913	WVIGGVSAHLFAVFQGLLKVLAGIDTNFTVTSK-A-S-DeD-GD--sAELYMfKWTTLLIPPTTLLIINLVGVVAGIS
>162955780	WVIGGVSAHLFAVFQGLLKVLAGIDTNFTVTSK-A-S-DeD-GD--sAELYMfKWTTLLIPPTTLLIINLVGVVAGIS
>561010122	WVIGGTSAHLFAVFQGLLKVLAGIDTNFTVTSK-A-T-DeD-GD--FAELYVfKWTSLLIPPTTVLIVNLVGIVAGvS
>410825282	WVIGGVSAHLFAVFQGLLKVLGGVDTNFTVTSK-S-A-D-D-AE--FGELYLfKWTTLLIPPTTLIILNMVGVVAGvS
>502132343	WVIGGTSAHLFAVFQGLLKVLAGIDTNFTVTSK-A-S-DeD-GD--FAELYVfKWTSLLIPPTTVLIVNLIGIVAGvS
>376315424	WVIGGTSAHLFAVFQGLLKVLAGIDTNFTVTSK-A-SdD-D-GD--FAELYVfKWTSLLIPPTTVLIINLVGIVAGvS
>381354084	WVIGGISAHLFAVFQGLLKVLAGIDTNFTVTSK-A-S-DeE-GD--FAELYLfKWTTLLIPPTTILIINLVGVVAGIS
>449448450	WVIGGVSSHLFALFQGLLKVLAGVSTNFTVTSK-A-A-D-D-GE--FSELYIfKWTSLLIPPTTLLIINIVGVIVGIS
>359476121	WVIGGTSAHLFAVFQGLLKVLAGIDTNFTVTSK-A-SdD-D-GD--FAELYVfKWTSLLIPPTTVLVVNLVGIVAGvS
>147775461	WVIGGTSAHLFAVFQGLLKVLAGIDTNFTVTSK-A-SdD-D-GD--FAELYVfKWTSLLIPPTTVLVVNLVGIVAGvS
>410825286	WVIGGVSAHLFAVFQGLLKVLGGVDTNFTVTSK-S-A-D-D-AE--FGELYLfKWTTLLIPPTTLIILNMVGVVAGvS
>66269686	WVIGGISAHLFAVFQGLLKVLAGIDTNFTVTSK-A-S-DeE-GD--FAELYMfKWTTLLIPPTTILIINLVGVVAGIS
>347953849	WVIGGVSAHLFAVFQGLLKVLAGIDTNFTVTSK-A-S-DeD-GD--FAELYMfKWTTLLIPPTTLLIINLVGVVAGIS
>325464705	WVIGGVSAHLFAVFQGLLKVLAGIDTNFTVTSK-A-S-DeD-GD--FAELYMfKWTTLLIPPTTLLIINLVGVVAGIS
>548845028	WVIGGTSAHLFAVFQGLLKVLAGIDTNFTVTSK-A-S-DeD-GD--FAELYVfKWTALLIPPTTVLLINMVGIVAGvS
>565471578	WVIGGTSAHLFAVFQGLLKVLAGIDTNFTVTSK-A-S-DeD-GD--FAELYVfKWTSLLIPPTTILLVNLVGIVAGvS
>502157997	WVIGGVSAHLFAVFQGLLKVLAGIDTNFTVTSK-A-S-DeD-GD--sAELYMfKWTTLLIPPTTLLIINLVGVVAGIS
>357457831	WVIGGVSAHLFAVFQGLLKVLAGIDTNFTVTSK-A-S-DeD-GD--sAELYMfKWTTLLIPPTTLLIINLVGVVAGIS
>47933334	WVIGGVSAHLFAVIQGLLKVLAGVDTNFTVTSK-A-S-D-EgGD--FAELYIIKWTALLIPPTTLLIINIVGVVAGIS
>502136908	WVIGGASSHLFALFQGLLKVLGGVDTNFTVTSK-A-A-D-D-GE--FSELYIfKWTSLLIPPTTLLIINIVGVVVGIS

>347953833	WVIGGVSAHLFAVFQGLLKVLAGIDTNFTVTSK-A-S-DeD-GD--FAELYMfKWTTLLIPPTTLLIINLVGVVAGIS
>15227094	WVIGGVSSHLFALFQGLLKVLAGVSTNFTVTSK-A-A-D-D-GE--FSELYIfKWTSLLIPPTTLLIINIVGVIVGvS
>325464697	WVIGGVSAHLFAVFQGLLKVLAGIDTNFTVTSK-A-S-DeD-GD--FAELYMfKWTTLLIPPTTLLIINLVGVVAGIS
>255548960	WVIGGTSAHLFAVFQGLLKVLAGIDTNFTVTSK-A-SdD-D-GD--FAELYVfKWTSLLIPPTTVIIVNLVGIVAGvS
>347953837	WVIGGVSAHLFAVFQGLLKVLAGIDTNFTVTSK-A-S-DeD-GD--FAELYMfKWTTLLIPPTTLLIINLVGVVAGIS
>442614085	WVIGGVSAHLFAVFQGLLKVLGGVDTNFTVTSK-S-A-D-D-AE--FGELYLfKWTTLLIPPTTLIILNMVGVVAGvS
>442614077	WVIGGVSAHLFAVFQGLLKVLGGVDTNFTVTSK-S-A-D-D-AE--FGELYLfKWTTLLIPPTTLIILNMVGVVAGvS
>530845216	WVIGGVSAHLFAVFQGLLKVLGGVDTNFTVTSK-S-A-D-D-AE--FGELYLfKWTTLLIPPTTLIILNMVGVVAGvS
>530845232	WVIGGVSAHLFAVFQGLLKVLGGVDTNFTVTSK-S-A-D-D-AE--FGELYLfKWTTLLIPPTTLIILNMVGVVAGvS
>40363755	WVIGGISAHLFAVFQGLLKVLAGIDTNFTVTSK-A-n-DeE-GD--FAELYMfKWTTLLIPPTTILIINMVGVVAGtS
>241740097	WVIGGTSAHLFAVFQGLLKVLAGIDTNFTVTSK-A-S-DeD-GD--FAELYIfKWTALLIPPTTVLVVNLIGIVAGvS
>241740088	WVIGGTSAHLFAVFQGLLKVLAGIDTNFTVTSK-A-S-DeD-GD--FAELYIfKWTALLIPPTTVLVVNLIGIVAGvS
>530845218	WVIGGVSAHLFAVFQGLLKVLGGVDTNFTVTSK-S-A-D-D-AE--FGELYLfKWTTLLIPPTTLIILNMVGVVAGvS
>530845242	WVIGGVSAHLFAVFQGLLKVLGGVDTNFTVTSK-S-A-D-D-AE--FGELYLfKWTTLLIPPTTLIILNMVGVVAGvS
>530845220	WVIGGVSAHLFAVFQGLLKVLGGVDTNFTVTSK-S-A-D-D-AE--FGELYLfKWTTLLIPPTTLIILNMVGVVAGvS
>475541489	WVIGGISAHLFAVFQGLLKVLAGIDTNFTVTSK-A-n-DeE-GD--FAELYMfKWTTLLIPPTTILIINMVGVVAGtS
>530845250	WVIGGVSAHLFAVFQGLLKVLGGVDTNFTVTSK-S-A-D-D-AE--FGELYLfKWTTLLIPPTTLIILNMVGVVAGvS
>67003915	WVIGGTSAHLFAVFQGLLKVLAGIDTNFTVTSK-A-G-DeD-GD--FAELYVfKWTSLLIPPTTVLIVNIIGIVAGvS
>340343839	WVIGGTSAHLFAVFQGLLKVLAGIDTNFTVTSK-A-G-DeD-GD--FAELYVfKWTSLLIPPTTVLIVNIIGIVAGvS
>460398994	WVIGGTSAHLFAVFQGLLKVLAGIDTNFTVTSK-A-n-DeD-GD--FAELYVfKWTTLLIPPTAILIVNLVGIVAGvS
>429326432	WVIGGTSAHLFAVFQGLLKVLAGIDTNFTVTSK-A-S-DeD-GD--FAELYVfKWTSLLIPPTTVIVLNMVGIVAGvS
>37725361	WVIGGTSAHLFAVFQGLLKVLAGIDTNFTVTSK-A-S-DeD-GD--FAELYVfKWTSLLIPPTTVIVLNMVGIVAGvS
>557106313	WVIGGTSAHLFAVFQGLLKVLAGIDTNFTVTSK-A-S-DeD-GD--FAELYIfKWTSLLIPPTTILLMNLVGIVAGvS
>66269688	WVIGGISAHLFAVFQGLLKVLAGIDTNFTVTSK-A-T-DeE-GD--FAELYMfKWTTLLIPPTTILIINLVGVVAGIS
>565375093	WVIGGVSAHLFAVFQGLLKVLAGVETNFTVTSK-S-G-D-D-eE--YAELYafKWTTLLIPPTTLLVINIIGVVAGIS
>460385657	WVIGGVSAHLFAVFQGLLKVLAGVETNFTVTSK-S-G-D-D-eE--YAELYafKWTTLLIPPTTLLVINIIGVVAGIS
>557113647	WVIGGTSAHLFAVFQGLLKVLAGIDTNFTVTSK-A-S-DeD-GD--FAELYIfKWTALLIPPTTVLLVNLIGIVAGvS
>565449881	WVIGGTSAHLFAVFQGLLKVLAGIDTNFTVTSK-A-S-DeD-GD--FAELYIfKWTALLIPPTTVLLVNLIGIVAGvS
>297798722	WVIGGTSAHLFAVFQGLLKVLAGIDTNFTVTSK-A-S-DeD-GD--FAELYIfKWTALLIPPTTVLLVNLIGIVAGvS
>183211890	WVIGGVSAHLFAVcQGLLKVLAGIDTNFTVTSK-A-S-DeD-GD--FtELYMfKWTTLLIPPTTLLIINLVGVVAGIS
>514765928	WVIGGTSAHLFAVFQGLLKVLAGIDTNFTVTSK-A-S-DeD-GD--FAELYVfKWTSLLIPPTTVLVINLVGMVAGIS
>514745194	WVIGGTSAHLFAVFQGLLKVLAGIDTNFTVTSK-A-S-DeD-GD--FAELYVfKWTSLLIPPTTVLVINLVGMVAGIS
>514745190	WVIGGTSAHLFAVFQGLLKVLAGIDTNFTVTSK-A-S-DeD-GD--FAELYVfKWTSLLIPPTTVLVINLVGMVAGIS
>162459760	WVIGGTSAHLFAVFQGLLKVLAGIDTNFTVTSK-A-S-DeD-GD--FAELYVfKWTSLLIPPTTVLVINLVGMVAGIS
>162460417	WVIGGTSAHLFAVFQGLLKVLAGIDTNFTVTSK-A-S-DeD-GD--FAELYVfKWTSLLIPPTTVLVINLVGMVAGIS
>413948740	WVIGGTSAHLFAVFQGLLKVLAGIDTNFTVTSK-A-S-DeD-GD--FAELYVfKWTSLLIPPTTVLVINLVGMVAGIS
>413948739	WVIGGTSAHLFAVFQGLLKVLAGIDTNFTVTSK-A-S-DeD-GD--FAELYVfKWTSLLIPPTTVLVINLVGMVAGIS
>242089723	WVIGGTSAHLFAVFQGLLKVLAGIDTNFTVTSK-A-S-DeD-GD--FAELYVfKWTSLLIPPTTVLVINLVGMVAGIS
>357466269	WVIGGTSAHLFAVFQGLLKVLAGIDTNFTVTSK-A-n-DeD-GD--FAELYVfKWTSLLIPPTTVLIVNLIGIVAGvS
>66269680	WVIGGTSAHLFAVFQGLLKVLAGIDTNFTVTSK-A-S-DeD-GD--FAELYVfKWTSLLIPPTTVLVINLVGMVAGIS
>66269678	WVIGGTSAHLFAVFQGLLKVLAGIDTNFTVTSK-A-S-DeD-GD--FAELYVfKWTSLLIPPTTVLVINLVGMVAGIS

>66269682	WVIGGTSAHLFAVFQGLLKVLAGIDTNFTVTSK-A-T-D-DeGD--FAELYVfKWTSLLIPPTTVLVINLVGIVAGvS
>115462377	WVIGGTSAHLFAVFQGLLKVLAGIDTNFTVTSK-A-S-DeD-GD--FAELYVfKWTSLLIPPTTVLVINLVGMVAGIS
>414871151	WVIGGVSAHLFAVFQGFLKVLGGVDTSFTVTSK-A-AgD-E-ADa-FGDLYLfKWTTLLVPPTTLIIINMVGIVAGvS
>162464424	WVIGGVSAHLFAVFQGFLKVLGGVDTSFTVTSK-A-AgD-E-ADa-FGDLYLfKWTTLLVPPTTLIIINMVGIVAGvS
>429326446	WVIGGVSAHLFAVFQGLLKVLAGVDTNFTVTSK-G-G-D-D-dE--sSELYafKWTTLLIPPTTLLIINLVGVVAGvS
>225690808	WVIGGISAHLFAVFQGLLKVLAGIDTNFTVTSK-A-n-DeE-GD--FAELYMfKWTTLLIPPTTILIINLVGVVAGIS
>475350006	WVIGGISAHLFAVFQGLLKVLAGIDTSFTVTSK-A-S-DeD-nD--FAELYMfKWTTLLIPPTTILIINLVGVVAGtS
>47933336	WVIGGVSAHLFAVvQGLLKVLAGIDTNFTVTSK-A-S-DeD-GD--FAELYLfKWTALLIPPTTLLVINIVGVVAGIS
>251766023	WVIGGISAHLFAVFQGLLKVLAGIDTNFTVTSK-A-n-DeE-GD--FAELYMfKWTTLLIPPTTILIINLVGVVAGIS
>530845244	WVIGGVSAHLFAVFQGLLKVLGGVDTNFTVTSK-T-A-D-D-AE--FGELYLfKWTTLLIPPTTLIILNMVGVVAGvS
>115456095	WVIGGISAHLFAVFQGLLKVLAGIDTSFTVTSK-A-S-DeE-GD--FAELYMfKWTTLLIPPTTILIINLVGVVAGIS
>381354082	WVIGGTSAHLFAVFQGLLKVLAGIDTNFTVTSK-A-T-D-DeGD--FSELYVfKWTSLLIPPTTVLVINLVGIVAGvS
>66269696	WVIGGTSAHLFAVFQGLLKVLAGIDTNFTVTSK-A-T-D-DeGD--FSELYVfKWTSLLIPPTTVLVINLVGIVAGvS
>357114977	WVIGGISAHLFAVFQGLLKVLAGIDTSFTVTSK-A-S-DeD-nD--FAELYMfKWTTLLIPPTTILIINLVGVVAGIS
>514725695	WVIGGISAHLFAVFQGLLKVLAGIDTNFTVTSK-A-n-DeE-GD--FAELYMfKWTTLLIPPTTILIINLVGVVAGIS
>224089205	WVIGGTSAHLFAVFQGLLKVLAGIDTNFTVTSK-A-S-DeD-GD--FAELYVfKWTSLLIPPTTVILLNMVGIVAGvS
>527200712	WVIGGTSAHLFAVFQGLLKVLAGIDTNFTVTSK-A-SdD-D-GD--FeELYVfKWTSLLIPPTTVLMLNMVGIVAGvS
>15236786	WVIGGTSAHLFAVFQGLLKVLAGIDTNFTVTSK-A-T-DeD-GD--FAELYIfKWTALLIPPTTVLLVNLIGIVAGvS
>357111188	WVIGGISAHLFAVFQGLLKVLAGIDTNFTVTSK-A-n-DeE-GD--FAELYMfKWTTLLIPPTTILIINLVGVVAGIS
>514816353	WVIGGVSAHLFAVFQGLLKVLGGVDTNFTVTSKgA-A-D-E-TDv-FGELYLfKWTTLLVPPTTLIIINMVGIVAGvS
>115482436	WVIGGVSAHLFAVFQGLLKVLGGVDTNFTVTSKaA-A-D-E-TDa-FGELYLfKWTTLLVPPTTLIIINMVGIVAGvS
>222612976	WVIGGVSAHLFAVFQGLLKVLGGVDTNFTVTSKaA-A-D-E-TDa-FGELYLfKWTTLLVPPTTLIIINMVGIVAGvS
>218184709	WVIGGVSAHLFAVFQGLLKVLGGVDTNFTVTSKaA-A-D-E-TDa-FGELYLfKWTTLLVPPTTLIIINMVGIVAGvS
>15224746	WVIGGTSAHLFAVFQGLLKVFAGIDTNFTVTSK-A-S-DeD-GD--FAELYVfKWTSLLIPPTTILLVNLVGIVAGvS
>386576412	WVIGGVSAHLFAVFQGLLKVLAGVDTNFTVTAK-A-A-E-D-TE--FGELYLfKWTTLLIPPTTLIILNtVGVVAGvS
>39726027	WVIGGISAHLFAVFQGLLKVLAGIDTSFTVTSK-A-S-DeD-nD--FAELYMfKWTTLLIPPTTILIINLVGVVAGtS
>326521154	WVIGGISAHLFAVFQGLLKVLAGIDTSFTVTSK-A-S-DeD-nD--FAELYMfKWTTLLIPPTTILIINLVGVVAGtS
>445566833	WVIGGTSAHLFAVFQGLLKVLAGIDTNFTVTSK-A-SdD-D-GE--FAELYVfKWTSLLIPPTTVLIINLVGIVAGvS
>557100561	WVIGGVSSHLFALFQGLLKVLAGVNTNFTVTSK-A-A-D-D-GE--FSELYIfKWTSLLIPPTTLFIINVIGVIVGvS
>356525568	WVIGGVSSHLFALFQGLLKVLAGVNTNFTVTSK-A-A-D-D-GE--FSELYIfKWTSLLIPPmTLLIMNIVGVVVGIS
>557556807	WVIGGISAHLFAVvQGLLKILAGIDTNFTVTSK-A-T-DeD-eE--FGELYsfKWTTLLIPPTTVLIINLVGVVAGIS
>3135611	WVIGGVSAHLFALFQGLLKVLAGVDTNFTVTSK-A-A-D-D-GE--FSDLYLfKWTSLLIPPmTLLIINVIGVIVGvS
>15238231	WVIGGVSAHLFALFQGLLKVLAGVDTNFTVTSK-A-A-D-D-GE--FSDLYLfKWTSLLIPPmTLLIINVIGVIVGvS
>237506883	WVIGGTSAHLFAVFQGLLKVLAGIDTNFTVTSK-A-S-DeD-GD--FAELYVfKWTSLIIPPTTVLVINLVGMVAGIS
>470106071	WVIGGASSHLFALFQGLLKVLAGVNTNFTVTTK-A-A-D-D-Ga--FSELYIfKWTSLLIPPTTLLIINLVGVVVGIS
>60299999	WVIGGVSAHLFAVFQGLLKVLGGVDTNFTVTAK-G-S-D-E-eDq-FGELYMfKWTTLLIPPTTLLIINLVSLVAGvS
>414873523	WVIGGISAHLFAVFQGLLKVLAGIDTSFTVTSK-A-T-DeE-GD--FAELYMfKWTTLLIPPTTILIINLVGVVAGIS
>297788772	WVIGGVSAHLFALFQGLLKVLAGVDTNFTVTSK-A-A-D-D-GE--FSDLYLfKWTSLLIPPTTLLIINVIGVIVGvS
>224096488	WVIGGASAHLFALFQGLLKVLAGVSTNFTVTSK-A-A-D-D-GE--FSELYLfKWTSLLIPPTTLLIMNIVGVVVGvS
>224096486	WVIGGASAHLFALFQGLLKVLAGVSTNFTVTSK-A-A-D-D-GE--FSELYLfKWTSLLIPPTTLLIMNIVGVVVGvS
>565392055	WVIGGTSAHLFAVFQGLLKVLAGIDTNFTVTSK-A-n-DeD-GD--FAELYVfKWTTLLIPPTAILIMNLVGIVAGvS

>514813108	WVIGGISAHLFAVFQGLLKVLAGIDTSFTVTSK-A-T-DeE-GD--FAELYMfKWTTLLIPPTTILIINLVGVVAGIS
>162460565	WVIGGISAHLFAVFQGLLKVLAGIDTSFTVTSK-A-T-DeE-GD--FAELYMfKWTTLLIPPTTILIINLVGVVAGIS
>356512789	WVIGGVSSHLFALFQGLLKVLAGVNTNFTVTSK-A-A-D-D-GE--FSELYIfKWTSLLIPPmTLLIMNIVGVVVGvS
>475588604	WVIGGTSAHLFAVFQGLLKVLAGIDTNFTVTSK-A-n-DeD-GD--FAELYVfKWTSLLIPPTTVLVINLVGMVAGIS
>475492890	WVIGGTSAHLFAVFQGLLKVLAGIDTNFTVTSK-A-n-DeD-GD--FAELYVfKWTSLLIPPTTVLVINLVGMVAGIS
>429326426	WVIGGVSSHLFALFQGLLKVLAGVSTNFTVTSK-G-A-D-D-GE--FSELYIfKWTSLLIPPTTLLIMNIVGVVVGvS
>357134454	WVIGGTSAHLFAVFQGLLKVLAGIDTNFTVTSK-A-n-DeD-GD--FAELYVfKWTSLLIPPTTVLVINLVGMVAGIS
>326518484	WVIGGTSAHLFAVFQGLLKVLAGIDTNFTVTSK-A-n-DeD-GD--FAELYVfKWTSLLIPPTTVLVINLVGMVAGIS
>224083850	WVIGGVSSHLFALFQGLLKVLAGVSTNFTVTSK-G-A-D-D-GE--FSELYIfKWTSLLIPPTTLLIMNIVGVVVGvS
>47078500	WVIGGVSSHLFALFQGLLKVLAGVSTNFTVTSK-G-A-D-D-GE--FSELYIfKWTSLLIPPTTLLIMNIVGVVVGvS
>39726035	WVIGGTSAHLFAVFQGLLKVLAGIDTNFTVTSK-A-n-DeD-GD--FAELYVfKWTSLLIPPTTVLVINLVGMVAGIS
>37725363	WVIGGVSSHLFALFQGLLKVLAGVSTNFTVTSK-G-A-D-D-GE--FSELYIfKWTSLLIPPTTLLIMNIVGVVVGvS
>359481817	WVIGGVSAHLFAVcQGLLKVLAGIDTNFTVTSK-A-S-DeE-GD--FAELYMfKWTTLLIPPTTLLIINLVGVVAGIS
>527205338	WVIGGVSSHLFALFQGLLKVLAGVNTNFTVTSK-G-G-D-D-GD--FSELYIfKWTSLLIPPTTLLILNIVGVLVGvS
>429326436	WVIGGTSAHLFAVFQGLLKVLAGIDTNFTVTSK-A-S-DeD-GD--FAELYVfKWTSLLIPPTTVILLNMMGIVAGvS
>224141885	WVIGGTSAHLFAVFQGLLKVLAGIDTNFTVTSK-A-S-DeD-GD--FAELYVfKWTSLLIPPTTVILLNMMGIVAGvS
>357111050	WVIGGVSAHLFAIFQGLLKVLAGVDTSFTVTSK-G-G-D-D-eE--FSELYtfKWTTLLIPPTTLLMLNFIGVVAGIS
>357111048	WVIGGVSAHLFAIFQGLLKVLAGVDTSFTVTSK-G-G-D-D-eE--FSELYtfKWTTLLIPPTTLLMLNFIGVVAGIS
>9622878	WVIGGTSAHLFAVFQGLLKVLAGIDTNFTVTSK-A-TdD-D-GD--FAELYVfKWTTLLIPPTTVLVINLVGIVAGvS
>429326444	WVIGGASAHLFALFQGLLKVLAGVSTNFTVTSK-A-A-D-D-GE--FSELYLfKWTSLLIPPTTLLIMNIVGVVVGvS
>530845246	WVIGGVSAHLFAVFQGLLKVLGGaDTNFTVTSK-T-A-D-D-AE--FGELYLfKWTTLLIPPTTLIILNMVGVVAGvS
>168045701	WVIGGVSAHLFALFQGLLKVFAGIDTNFTVTSK-q-A-E-D-eD--FAELYMIKWTALLIPPTTLIVINMIGVVAGIS
>297739672	WVIGGVSAHLFAVcQGLLKVLAGIDTNFTVTSK-A-S-DeE-GD--FAELYMfKWTTLLIPPTTLLIINLVGVVAGIS
>66269690	WVIGGISAHLFAVFQGLLKVLAGIDTSFTVTSK-A-S-DeE-GD--FtELYMfKWTTLLIPPTTILIINLVGVVAGIS
>514776033	WVIGGTSAHLFAVFQGLLKVLAGIDTNFTVTSK-A-T-DeE-GD--FSELYVfKWTSLLIPPTTVLVINLVGIVAGvS
>502139941	WVIGGASSHLFALFQGLLKVLAGVDTNFTVTSK-A-A-D-D-GE--FSELYVfKWTSLLIPPmTLLIMNIVGVIVGvS
>357519771	WVIGGASSHLFALFQGLLKVLAGVDTNFTVTSK-A-A-D-D-GE--FSELYVfKWTSLLIPPmTLLIMNIVGVIVGvS
>213522383	WVIGGTSAHLFAVFQGLLKVLAGIDTNFTVTSK-A-T-DeE-GD--FSELYVfKWTSLLIPPTTVLVINLVGIVAGvS
>530845230	WVIGGVSAHLFAVFQGLLKVpGGVDTNFTVTSK-S-A-D-D-AE--FGELYLfKWTTLLIPPTTLIILNMVGVVAGvS
>565435957	WVIGGVSAHLFAVFQGLLKVLfGVDTNFTVTSKgA-S-D-E-ADe-FGDLYLfKWTTLLIPPTTLIILNMVGVVAGvS
>27462651	WVIGGVSAHLFAVFQGLLKVLfGVDTNFTVTSKgA-S-D-E-ADe-FGDLYLfKWTTLLIPPTTLIILNMVGVVAGvS
>241740128	WVIGGVSAHLFAVFQGLLKVLfGVDTNFTVTSKgA-S-D-E-ADe-FGDLYLfKWTTLLIPPTTLIILNMVGVVAGvS
>241740121	WVIGGVSAHLFAVFQGLLKVLfGVDTNFTVTSKgA-S-D-E-ADe-FGDLYLfKWTTLLIPPTTLIILNMVGVVAGvS
>297795005	WVIGGVSAHLFAVFQGLLKVLfGVDTNFTVTSKgA-S-D-E-ADe-FGDLYLfKWTTLLIPPTTLIILNMVGVVAGvS
>30694433	WVIGGVSAHLFAVFQGLLKVLfGVDTNFTVTSKgA-S-D-E-ADe-FGDLYLfKWTTLLIPPTTLIILNMVGVVAGvS
>95020352	WVIGGTSAHLFAVFQGLLKVLAGIDTNFTVTSK-A-SdD-D-GE--FAELYVfKWTSLLIPPTTVLIINLVGIVAGvS
>242205330	WVIGGTSAHLFAVFQGLLKVLAGIDTNFTVTSK-A-SdD-D-GD--FAELYVfKWTSLLIPPTTVLIINLVGIVAGvS
>414588936	WVIGGISAHLFAVFQGLLKVLAGIDTNFTVTSK-A-S-DeD-GD--FAELYMfKWTTLLIPPTTILIINLVGVVAGIS
>9758562	WVIGGVSAHLFAVFQGLLKVLfGVDTNFTVTSKgA-S-D-E-ADe-FGDLYLfKWTTLLIPPTTLIILNMVGVVAGvS
>557104262	WVIGGVSAHLFAVFQGLLKVLfGVDTNFTVTSK-G-AtD-E-ADe-FGDLYLfKWTTLLIPPTTLIILNMVGVVAGvS
>386576416	WVIGGISAHLFAVvQGLLKILAGIDTNFTVTSK-A-T-D-D-dD--FGELYafKWTTLLIPPTTILIINLVGVVAGIS

>326493686	WVIGGISAHLFAVFQGLLKVLAGIDTNFTVTSK-A-n-DeE-GD--FAELYMfKWTTLLIPPTTILIINMVGVVAGtS
>296939593	WVIGGISAHLFAVFQGLLKVLAGIDTNLTVTSK-A-n-DeE-GD--FAELYMfKWTTLLIPPTTILIINLVGVVAGIS
>242032585	WVIGGISAHLFAVFQGLLKVLAGIDTSFTVTSK-A-T-DeE-GD--FAELYMfKWTTLLIPPTTILIINLIGVVAGtS
>376315422	WVIGGVSSHLFALFQGLLKVLAGVSTSFTVTSK-A-A-D-D-GE--FSELYLfKWTSLLIPPTTLLVINIIGVVVGIS
>110740025	WVIGGVSAHLFAVFQGILKVLAGIDTNFTVTSK-A-S-DeD-GD--FAELYLfKWTTLLIPPTTLLIVNLVGVVAGvS
>18875454	WVIGGVSAHLFAVFQGFLKMLAGVDTNFTVTAK-A-A-D-D-qE--FGELYMIKWTTVLIPPTTLLVLNLVGVVAGFS
>251766021	WVIGGTSAHLFAVFQGLLKVLAGIDTNFTVTSK-A-T-DeE-GD--FSELYVfKWTSLIIPPTTVLVINLVGIVAGvS
>270486534	WVIGGTSAHLFAVFQGLLKVLAGIDTNFTVTSK-A-T-DeE-GD--FSELYVfKWTSLIIPPTTVLVINLVGIVAGvS
>357158469	WVIGGVSAHLFAVIQGLLKVLAGIDTNFTVTSK-A-T-g-D-eDdeFAELYtfKWTTLLIPPTTLLIINIIGVVAGIS
>541135555	WVIGdVSAHLFAVcQGLLKVLAGIDTNFTVTSK-A-S-DeD-GD--FtELYMfKWTTLLIPPTTLLIINLVGVVAGIS
>429326442	WVIGGASSHLFALFQGLLKVLAGVNTNFTVTSK-A-A-D-D-GE--FSELYLfKWTSLLIPPmTLLIINIIGVVVGIS
>224082476	WVIGGASSHLFALFQGLLKVLAGVNTNFTVTSK-A-A-D-D-GE--FSELYLfKWTSLLIPPmTLLIINIIGVVVGIS
>326492019	WVIGGTSAHLFAVFQGLLKVLAGIDTNFTVTSK-A-n-DeD-GD--FAELYVfKWTSLLIPPTTVLVINLVGMVAGIS
>297825603	WVIGGTSAHLFAVFQGLLKVFAGIDTNFTVTSK-A-S-DeD-GD--FAELYVfKWTSLLIPPTTILLVNLVGIVVGvS
>326513678	WVIGGISAHLFAVFQGLLKVLAGIDTNFTVTSK-A-n-DeE-GD--FAELYMfKWTTLLIPPTTILIINMVGVVTGtS
>47078494	WVIGGASSHLFALFQGLLKVLAGVNTNFTVTSK-A-A-D-D-GE--FSELYLfKWTSLLIPPmTLLIINIIGVVVGIS
>224066625	WVIGGASAHLFALFQGLLKVLAGVNTNFTVTSK-A-A-D-D-GE--FSDLYLfKWTSLLIPPmTLLIINIIGVVVGIS
>462409578	WVIGGASSHLFALFQGLLKVLAGVNTNFTVTSK-A-A-D-D-Ga--FSELYIfKWTALLIPPmTLLIINIVGVVVGIS
>403322650	WVIGGVSAHLFAVvQGLLKVLAGIDTNFTVTSK-A-T-D-D-dD--FGELYafKWTTLLIPPTTILIINLVGVVAGvS
>33413762	WVIGGVSAHLFAVFQGLLKVLAGVDTNFTVTAK-x-x-E-D-iE--xGELYLfKWTTLLIPPTTLIILNMVGVVAGvS
>414589525	WVIGGVSAHLFAVvQGLLKVLAGIDTNFTVTSK-A-T-g-D-eDdeFAELYafKWTTLLIPPTTLLIINIIGVVAGIS
>403323092	WVIGGVSAHLFAVvQGLLKVLAGIDTNFTVTSK-A-T-D-D-dD--FGELYafKWTTLLIPPTTILIINLVGVVAGvS
>403323052	WVIGGVSAHLFAVvQGLLKVLAGIDTNFTVTSK-A-T-D-D-dD--FGELYafKWTTLLIPPTTILIINLVGVVAGvS
>475617494	WVIGGVSAHLFAVIQGLLKVLAGIDTNFTVTSK-A-T-g-D-eDdeFAELYafKWTTLLIPPTTLLVINIIGVVAGIS
